# Promoter Methylation Leads to Hepatocyte Nuclear Factor 4A Loss and Pancreatic Cancer Aggressiveness

**DOI:** 10.1016/j.gastha.2024.04.005

**Published:** 2024-04-24

**Authors:** Maria Hatziapostolou, Marina Koutsioumpa, Abed M. Zaitoun, Christos Polytarchou, Mouad Edderkaoui, Swapna Mahurkar-Joshi, Jayakumar Vadakekolathu, Daniel D'Andrea, Anna Rose Lay, Niki Christodoulou, Thuy Pham, Tung-On Yau, Christina Vorvis, Suchit Chatterji, Stephen J. Pandol, George A. Poultsides, David W. Dawson, Dileep N. Lobo, Dimitrios Iliopoulos

**Affiliations:** 1 Department of Biosciences, John van Geest Cancer Research Centre, Centre for Systems Health and Integrated Metabolic Research, School of Science and Technology, Nottingham Trent University, Nottingham, UK; 2 Vatche and Tamar Manoukian Division of Digestive Diseases, Center for Systems Biomedicine, David Geffen School of Medicine, University of California at Los Angeles, Los Angeles, California; 3 Department of Cellular Pathology, Nottingham Digestive Diseases Centre and NIHR Nottingham Biomedical Research Centre, Nottingham University Hospitals and University of Nottingham, Queen’s Medical Centre, Nottingham, UK; 4 Departments of Medicine and Biomedical Sciences, Cedars-Sinai Medical Center, Los Angeles, California; 5 Department of Pathology and Laboratory Medicine, David Geffen School of Medicine, University of California, Los Angeles, California; 6 Jonsson Comprehensive Cancer Center, David Geffen School of Medicine, University of California, Los Angeles, California; 7 Department of Surgery, Stanford University School of Medicine, Stanford, California; 8 Nottingham Digestive Diseases Centre and NIHR Nottingham Biomedical Research Centre, Nottingham University Hospitals and University of Nottingham, Queen’s Medical Centre, Nottingham, UK; 9 MRC Versus Arthritis Centre for Musculoskeletal Ageing Research, School of Life Sciences, Queen’s Medical Centre, University of Nottingham, Nottingham, UK

**Keywords:** Pancreatic Cancer, DNA Methylation, Epigenetics, HNF4A

## Abstract

**Background and Aims:**

Decoding pancreatic ductal adenocarcinoma heterogeneity and the consequent therapeutic selection remains a challenge. We aimed to characterize epigenetically regulated pathways involved in pancreatic ductal adenocarcinoma progression.

**Methods:**

Global DNA methylation analysis in pancreatic cancer patient tissues and cell lines was performed to identify differentially methylated genes. Targeted bisulfite sequencing and in vitro methylation reporter assays were employed to investigate the direct link between site-specific methylation and transcriptional regulation. A series of in vitro loss-of-function and gain-of function studies and in vivo xenograft and the KPC (*LSL-Kras*^*G12D/+*^; *LSL-Trp53*^*R172H/+*^; *Pdx1-Cre*) mouse models were used to assess pancreatic cancer cell properties. Gene and protein expression analyses were performed in 3 different cohorts of pancreatic cancer patients and correlated to clinicopathological parameters.

**Results:**

We identify Hepatocyte Nuclear Factor 4A (HNF4A) as a novel target of hypermethylation in pancreatic cancer and demonstrate that site-specific proximal promoter methylation drives HNF4A transcriptional repression. Expression analyses in patients indicate the methylation-associated suppression of HNF4A expression in pancreatic cancer tissues. In vitro and in vivo studies reveal that HNF4A is a novel tumor suppressor in pancreatic cancer, regulating cancer growth and aggressiveness. As evidenced in both the KPC mouse model and human pancreatic cancer tissues, HNF4A expression declines significantly in the early stages of the disease. Most importantly, HNF4 loss correlates with poor overall patient survival.

**Conclusion:**

HNF4A silencing, mediated by promoter DNA methylation, drives pancreatic cancer development and aggressiveness leading to poor patient survival.

## Introduction

Pancreatic ductal adenocarcinoma (PDAC), the predominant form of pancreatic cancer,^1^ is a fatal disease predicted to be ranked as the second cause of death by cancer in the United States.^2^ Major clinical challenges in the management of PDAC extend from screening and diagnosis to the therapeutic spectrum. The disease is usually diagnosed at an advanced stage, so the optimal treatment strategies available to date are not suitable for > 80% of patients who at the time of the diagnosis have either locally advanced or a metastatic PDAC.^3^ Significant therapeutic challenges for the advanced disease, resistance to radiotherapy and chemotherapy, are reflected by the low overall 5-year survival rate of 10%.

In recent years, much has been learned about the genetic events that characterize initiation and progression of PDAC. PDAC usually arises from precursor lesions, called pancreatic intraepithelial neoplasias (PanINs), and develops through a stepwise acquisition of genetic alterations into an invasive adenocarcinoma.^1^ Activating *KRAS* point mutations and telomere shortening are of the earliest events, followed by *CDKN2A* and *CDKN1A* inactivating mutations. At later stages of carcinogenesis, 2 critical tumour suppressors are inactivated, *TP53* and *SMAD4*.^4^^,^^5^ However, efforts to decode PDAC heterogeneity and the consequent guiding of therapeutic selection remain a challenge.^6^, ^7^, ^8^, ^9^ Epigenetic profiling can identify PDAC subtypes and distinguish metastatic lesions from primary tumours, within the same patient.^10^^,^^11^ The epigenomic nature of PDAC heterogeneity shows that genomic, transcriptomic, and epigenomic data integration is a premise for the identification of novel therapeutic targets.

Dysfunction and/or deregulation of nuclear receptors in cancer and the nature of their activation, by low-molecular mass ligands, render them attractive drug targets for cancer therapeutics.^12^ Hepatocyte Nuclear Factor 4A (HNF4A or NR2A1) is a highly conserved member of the nuclear receptor superfamily.^13^^,^^14^ It is highly expressed in the epithelium of pancreas, liver, and colon, controlling the transcription for a plethora of genes and regulating diverse downstream biological processes.^15^ HNF4A expression is highly complex, regulated at multiple levels: transcriptional (including promoter regulation), post-transcriptional (miRNA-mediated), and post-translational (protein phosphorylation and degradation).^16^

In this study, we have performed a global DNA methylation analysis, in pancreatic cancer patient tissues and cell lines, and identified HNF4A as a target of hypermethylation. Integration of HNF4A cancer-specific methylation and expression data identified promoter methylation as the driving force of HNF4A suppression. Targeted bisulfite sequencing and in vitro methylation reporter assays provided a direct link between specific promoter cytosine-guanine site methylation and HNF4A transcriptional regulation. In vitro loss and gain of functional assays uncovered a tumour suppressive role for HNF4A, further supported in mouse models of pancreatic cancer. Finally, this study using tissues from 3 different cohorts of patients demonstrates that HNF4A loss is an early event in pancreatic cancer and correlates with poor patient survival.

## Materials and Methods

### Cell Culture, Transient Transfection, and Lentiviral Transduction

All pancreatic cancer cell lines (AsPC-1, BxPC-3, Capan-1, Capan-2, CFPAC-1, HPAF-II, MIA PaCa-2, and PANC-1) were purchased from ATCC. AsPC-1 and BxPC-3 were grown in RPMI-1640, Capan-2 in McCoy’s 5a, CFPAC-1 in Iscove’s modified Dulbecco’s medium, HPAF-II in MEM-a, and PANC-1 in DMEM, all supplemented with 10% FBS. Capan-1 were grown in Iscove’s modified Dulbecco’s medium supplemented with 20% FBS and MIA PaCa-2 in DMEM supplemented with 10% FBS and 2.5% horse serum. HEK293T cells were purchased from ATCC and grown in DMEM supplemented with 10% FBS. All cell culture reagents were purchased from Life Technologies.

Cells were transfected with siRNAs for HNF4A (Table A1) using Lipofectamine RNAiMax transfection reagent (13778150, Life Technologies). Lentiviruses were produced in HEK293T cells transfected with the packaging and expression constructs using Fugene 6 (E2691, Promega). Cells were infected with the viruses using DEAE Dextran (Sigma-Aldrich) and selected with puromycin (Sigma-Aldrich).

### Cloning

HNF4A sequence, tagged with MYC-Flag, was obtained from HNF 4 alpha (HNF4A) (HNF4alpha1, NM_178849) Human Tagged ORF Clone Lentiviral Particle (RC214914, Origene) and cloned into the pLenti CMV/TO Puro DEST vector (17293, Addgene). Cloning was achieved by using the pENTR/D-TOPO Cloning Kit (K2435), Gateway LR Clonase II Enzyme mix (11791, Life Technologies), and the primers F, 5′-CACCATGCGACTCTCCAAAACCCTC-3′ and R, 5′-TTAAACCTTATCGTCGTCATCCTTGT-3′. Sequences containing the shRNAs for HNF4A (Table A1) were annealed and cloned into the pLKO.1 puro (8453, Addgene) vector according to the Janes Lab protocol (http://bme.virginia.edu/janes/protocols/pdf/Janes_shRNAcloning.pdf).

### Reverse Transcription qPCR

Total RNA, from cells and tissues, was purified with TRIZOL (15596026, Invitrogen), AllPrep DNA/RNA FFPE kit (80234, Qiagen), or the RNeasy Mini Kit (74104, Qiagen) according to manufacturer’s instructions. RNA was reverse transcribed to cDNA using the iSCRIPT RT Supermix (1708841, Bio-Rad), and qPCR analysis was performed using the iTaq Universal SYBR Green Supermix (1725124, Bio-Rad). The primer sequences (Tables A1 and A2) used for qPCR were designed using the NCBI Nucleotide Database (http://www.ncbi.nlm.nih.gov/nuccore), Primer3 v.0.4.0 (http://bioinfo.ut.ee/primer3-0.4.0), and UCSC In-Silico PCR (http://genome.ucsc.edu/cgi-bin/hgPcr).

### Immunohistochemistry

For immunofluorescence staining, pancreatic cancer TMAs (PA483e, US Biomax) were subjected to deparaffinization, rehydration, and antigen unmasking as described in Supplementary Methods. Blocking was accomplished by incubating the slides in Buffer-W (NanoString) at room temperature for 1 hour. Then, slides were incubated with HNF4A antibody at the dilution of 1:100, for 1 hour at room temperature. For HNF4A detection, Alexa Fluor 647-conjugated Goat Anti-Rabbit IgG (ab150083, Abcam, 1:200) was applied to the slides and incubated for 1 hour. The slides were subjected to 3 washes with TBS-T and counterstained with Syto-13 nuclear stain (S7575, Invitrogen), for 1 hour at room temperature. Images were captured using the NanoString GeoMx Digital Spatial Profiling platform. For DAB immunostaining, see Supplementary Methods.

### Targeted Bisulfite Sequencing and Data Analysis

Next-generation sequencing for the evaluation of DNA methylation, at the level of single nucleotide resolution, has been conducted by Zymo Research Corporation, Irvine, CA. Cells were treated with or without 1 μM of 5-Aza-CdR (A3656, Sigma-Aldrich), for 48 hours and genomic DNA was isolated. Assays were designed targeting CpG sites in the specified regions of interest, listed in Table A2. Primers were generated with Rosefinch, Zymo Research’s proprietary sodium bisulfite converted DNA-specific primer design tool. Samples were bisulfite converted using the EZ DNA Methylation-LightningTM Kit (D5030, Zymo Research) according to the manufacturer’s instructions. Multiplex amplification of all samples, using the respective specific primer pair and the Fluidigm Access ArrayTM System, was performed according to the manufacturer’s instructions. The resulting amplicons were pooled for harvesting and subsequent barcoding according to the Fluidigm instrument’s guidelines. After barcoding, samples were purified using ZR-96 DNA Clean & Concentrator-5 (D4023, Zymo Research) and then prepared for massively parallel sequencing using a MiSeq V2 300bp Reagent Kit and paired-end sequenced.

Sequence reads were identified using standard Illumina base-calling software and then analysed using a Zymo Research proprietary analysis pipeline. Low-quality nucleotides and adapter sequences were trimmed off during analysis QC. Paired-end alignment was used as default. Index files were constructed using the bismark_genome_preparation command and the entire reference genome. The nondirectional parameter was applied while running Bismark. All other parameters were set to default. Nucleotides in primers were trimmed off from amplicons during methylation calling.

### In Vitro Methylated Reporter Assay

5′ NsiI and 3′ HindIII restriction sites were introduced into a 250bp fragment of the proximal HNF4A promoter region (containing the 8 CpG sites of interest), through PCR reaction, using genomic DNA as the template, Q5 high-fidelity DNA polymerase (M0491, New England Biolabs), and the primers F, 5′-ATATGCATGTCATGATGCCTGCCTTGTA-3′ and R, 5′-AAAAGCTTAAACCGTCCTCTGGGAAGAT-3′. Following a 2% agarose gel electrophoresis, the resulting PCR product was extracted using QIAquick Gel Extraction Kit (28704, Qiagen). The purified PCR product was subcloned into the pCpGfree-basic-*Lucia* reporter plasmid (pcpgf-basIc, InvivoGen), that codes for *Lucia*-secreted luciferase variant, following digestion with *NsiI* and *HindIII* restriction enzymes (R0127S and R0104S, respectively, New England Biolabs) and ligation through T4 DNA ligase (M0202S, New England Biolabs). Sequence was verified by DNA sequencing. Plasmids were methylated using *M.SssI* (M0226S, New England Biolabs) or *M.HpaII* (M0214S, M0214S) CpG methyltransferases according to manufacturer’s instructions. Briefly, 4 μg of the HNF4A promoter-Lucia reporter construct were incubated with 16 U of *M.SssI* or *M.HpaII* CpG methyltransferases and 640 μM S-Adenosylmethionine (B9003S, NEB) at 37 °C for 4 hours. The reaction was stopped by heating at 65 °C for 20 minutes. The unmethylated control construct was treated as above but without CpG methyltransferases or S-Adenosylmethionine.

Next, HEK293T cells were transiently transfected with unmethylated, *M.SssI* or *M.HpaII* methylated reporter constructs by using Fugene 6 transfection reagent (E2691, Promega). Equal number of cells was seeded into 96-well plates and luciferase activity of the Lucia reporter was measured, in the cell supernatant, at 48 and 72 hours following the transfection, using the QUANTI-*Luc* assay reagent (rep-qlc1, InvivoGen). In each experiment, the cells were co-transfected with pCMV-*Cypridina Luc* Vector (16150, ThermoFisher Scientific), which secretes a variant of *Cypridina* luciferase, as a normalization. Luminescence induced by *Cypridina* luciferase in the supernatant of cells was determined using the Pierce Cypridina luciferase lash assay kit (16168, ThermoFisher Scientific). Reporter activity was normalized by calculating the ration of *Lucia* to *Cypridina* activity.

### Spheroid Assay

Spheroids were generated using different approaches: the ultra-low attachment plate and the hanging drop method. For the ultra-low attachment plate method (174925, Thermo Scientific), 1000 PANC-1 cells were added in 200 μL suspension, per well, and centrifuged at 1500 rpm for 10 minutes at room temperature. For the hanging drop method, 20 μL drops containing 30,000 cells were pipetted onto the lid of 100 mm dishes and were inverted over dishes containing 10 mL PBS. All the cultures were incubated under standard conditions for 7 days, partially replacing the media every 3 days. Formation of spheroids was monitored daily and images were obtained by using a phase contrast microscope at a 10× magnification (EVOS XL Core Imaging System).

### Subcutaneous Xenograft Mouse Model

MIA PaCa-2 and PANC-1 cells were stably transduced with HNF4A or green fluorescent protein (GFP) lentiviral expressing constructs. HPAF-II cells were transduced with shRNA against HNF4A or the respective control. 4 × 10^6^ cells were injected subcutaneously in the right flank of NOD-SCID mice and tumor growth was monitored every 5–8 days for a total period of 4 weeks. Experiments were performed at UCLA David Geffen School of Medicine (CA, USA) and the John van Geest Cancer Research Centre at NTU (Nottingham, UK) according to guidelines and protocols approved by the respective animal research committees (ARC # 2015-049-01 and PD746B3FD, respectively).

### KPC (LSL-Trp53^R172H/+^, LSL-Kras^G12D/+^, Pdx 1-Cre) Mouse Model

The KPC mouse model has been described previously.^17^^,^^18^ Briefly, KPC mice derived from conditional *LSL-Trp53*^*R172H/+*^, *LSL-Kras*^*G12D/+*^, and *Pdx-1-Cre* strains were interbred to obtain *LSL-Kras*^*G12D/+*^; *LSL-Trp53*^*R172H/+*^; *Pdx-1-Cre* triple mutant animals. KPC mice mimic the human disease by developing PanIn lesions from the age of 4–6 weeks followed by a full cancer characterized by strong inflammatory response and fibrosis by the age of 2 months. PanIN and PDAC tissues were collected from male and female mice at the age of 3.5–5 months, fixed in formalin, and embedded in paraffin. All animal procedures were conducted according to the Cedars-Sinai (Los Angeles, CA, USA) Institutional Animal Care and Use Committee guidelines and regulations (IACUC protocol: #7661), and there was adequate representation of male and female mice.

### Patient Tissue Samples and Data Analysis

*Stanford Cohort*: Human pancreatic tissues were provided by the Department of Surgery at Stanford University (IRB#14-000217, Stanford, CA, USA). Genomic DNA and total RNA were extracted from 11 control (noninvolved pancreas) and 20 pancreatic adenocarcinoma tissues and were used for global DNA methylation and gene expression analyses (through RT-qPCR and gene expression microarray), respectively. *Commercial Tissue Microarray (TMA)*: Pancreas adenocarcinoma with pancreas tissue array was used to assess HNF4A expression through immunohistochemistry (PA1001c, US Biomax). *UCLACohort*: The University of California, Los Angeles stages I and II pancreatic cancer TMA^19^ consisted of 145 occurrences of AJCC stage I or II pancreatic adenocarcinoma from the University of California, Los Angeles Department of Pathology and Laboratory Medicine archives, which represented patients who underwent gross resection of tumor at University of California, Los Angeles Medical Center (Los Angeles, CA, USA). The TMA was used to assess HNF4A expression through immunohistochemical analysis. *QMC Cohort*: Human pancreatic tissues were obtained from the Division of Gastrointestinal Surgery at Queen’s Medical Centre (Protocol 18/HRA/0292, QMC, University of Nottingham, Nottingham, UK). HNF4A immunohistochemical analysis was performed in 38 control (noninvolved pancreas) and 168 pancreatic adenocarcinoma tissues of stages I–IV.

In UCLA and QMC cohort, 3 separate cores for each tumor were independently scored for HNF4A nuclear positivity by a blinded observer using semi-quantitative histoscores, representing the product of nuclear staining intensity (0–3) and percentage of tumor nuclei staining at that intensity (0–100). Histologic evaluation was performed in the respective Departments of Pathology (UCLA and QMC). For dichotomization, each tumor was assigned into either a low-level or high-level staining group based on its average histoscore. Survival estimates were generated with OriginPro, using the Kaplan-Meier method and compared using log-rank tests. Univariate and multivariate Cox proportional hazards models were performed in the R statistical computing (R version 3.4.3) environment and used to test statistical independence and significance of multiple predictors with backward selection performed using the Akaike Information Criterion.

In all cases, all work was performed with appropriate institutional review board approvals and there was adequate representation of tissues from male and female patients.

### Statistical and Bioinformatic Analysis

Graph generation, fold changes, and statistical significance were assessed using OriginPro. Statistical differences were calculated using unpaired *t*-test. Significance differences were considered when ∗*P* < .05, ∗∗*P* < .01, and ∗∗∗*P* < .001. Pathway and network analyses were performed using the MetaCore and Ingenuity Pathway Analysis softwares.

### Data Availability Statement

All authors had access to the study data and had reviewed and approved the final manuscript. Bisulfite sequencing and 450K Methylation array data have been deposited to the Gene Expression Omnibus and Zenodo databases and are available under accession number GSE85961 and https://doi.org/10.5281/zenodo.10138080, respectively. All data, generated by the authors in this study, are available upon request from the corresponding author.

Human Methylation 450K Array, cell growth, anchorage-independent cell growth, and invasion assays are described in the Supplementary Methods.

## Results

### HNF4A Is Epigenetically Suppressed in Pancreatic Cancer

**Figure 1:**
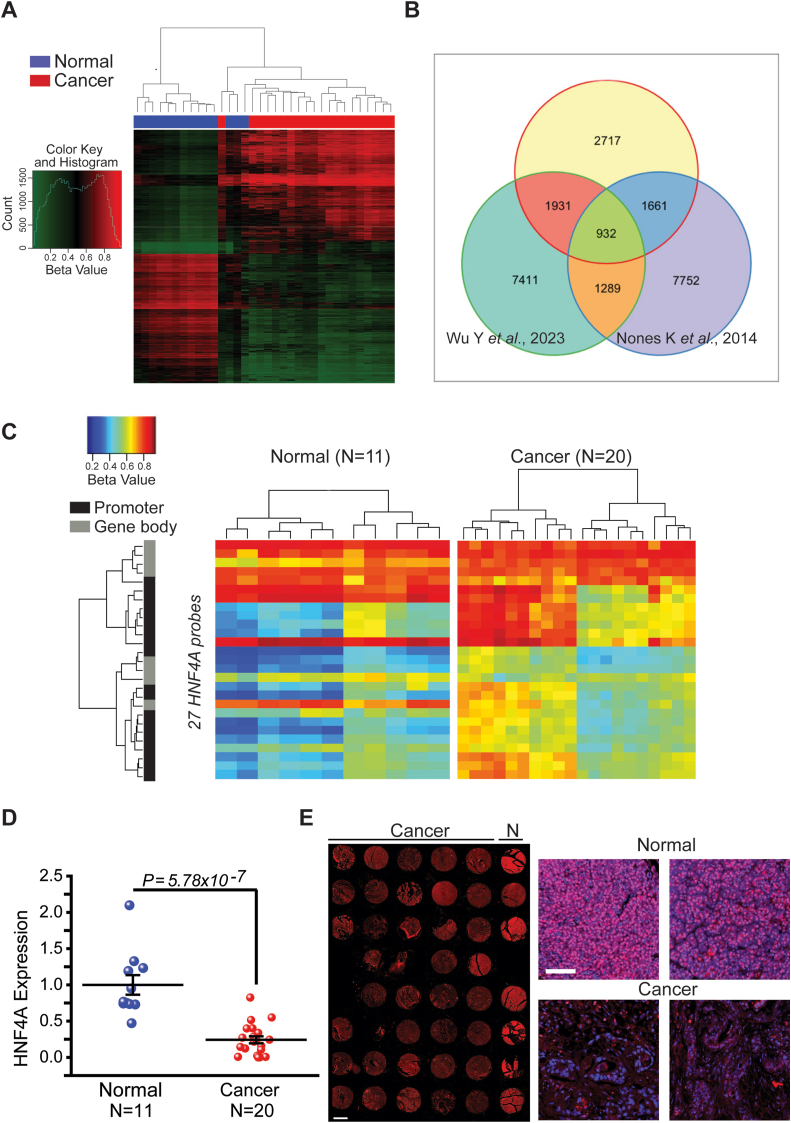
HNF4A is methylated and suppressed in pancreatic cancer. (A) Global DNA methylation analysis (Illumina Human Methylation 450K array) in 20 human pancreatic cancer and 11 normal tissues (Stanford University Medical Center, USA). Heatmap of methylation beta values for the top 7242 differentially regulated loci in cancer vs control (*P* < .05, FC ≥ 1.5). (B) Venn diagram showing 2717 unique methylation sites identified in our study. (C) Heatmap of methylation beta values across the HNF4A locus. The HNF4A promoter area with increased DNA methylation (high beta values) corresponds to probes covering the proximal promoter area (from −200 nt to −3 nt, TSS200). (D) HNF4A expression as assessed by RT-qPCR, in pancreatic cancer and control tissues. Expression was normalized to GAPDH and β-actin levels and results were expressed as mean ± SEM compared to control tissues (set as 1). (E) Immunohistochemical analysis for HNF4A in normal (N) and pancreatic cancer tissues (red, HNF4A; blue, nuclei staining). Scale bar: 1 mm and 100 μm (left and right panel, respectively).

Our goal was to determine how DNA methylation affects molecular mechanisms that drive pancreatic cancer. We firstly characterized global DNA methylation patterns in human pancreatic tissues (Stanford cohort, USA). We employed the Illumina Human Methylation 450K bead chip, in 20 human pancreatic cancer and 11 uninvolved tissues. Methylation analysis revealed a total of 26,307 CpG sites, differentially methylated in cancer. Of these, 18,425 (70%) were hypermethylated and 7882 (30%) hypomethylated. Given the negative regulatory relationship between promoter methylation and gene expression, we interrogated methylation in the promoter gene areas. We identified 9234 hypermethylated and 3794 hypomethylated gene promoter areas. Of these, 7241 were associated with a robust (*P* < .05, FC ≥ 1.5) differential promoter methylation (Figure 1A) and were used for pathway analysis. Comparison with previously published datasets,^20^^,^^21^ using the Illumina Human Methylation 450K chip, revealed 2717 novel methylation sites (Figure 1B and Supplementary Data-DNA methylation).

One differentially methylated network identified involves members of the HNF family (Figure A1): HNF4A (a nuclear receptor), HNF1A (a homeodomain protein), and members of the onecut homeobox family (ONECUT). HNFs are well described transcriptional regulators required for the normal function of the liver and pancreas.^22^ Herein, HNF4A was pinpointed as one of the aberrantly hypermethylated genes in primary pancreatic cancers (Figure 1C and Figures A2–A4). Using 27 different probes that span across the HNF4A locus, from the distal promoter to the 3′-UTR region, we were able to determine differences in the scale and distribution of HNF4A DNA methylation pattern. HNF4A displayed cancer-specific hypermethylation in the promoter and gene body regions (Figure 1C).

HNF4A locus susceptibility to methylation, in pancreatic cancer, indicated possible causative mediation effects on gene expression. RT-qPCR analysis revealed a significant reduction (∼5 fold) in HNF4A mRNA levels (Figure 1D). In support of these data, immunohistochemical analysis showed HNF4A suppression in pancreatic cancer. Additionally, we found that HNF4A is predominantly detected in the nuclear compartment of ductal epithelium and the surrounding acinar tissue (Figure 1E and Figure A5).

Ingenuity and MetaCore pathway analysis confirmed crucial pathways identified in similar studies, such as the axon guidance^20^ (Figure A6) and identified key cancer signaling pathways that are known to be genetically altered in pancreatic cancer (Figures A7–A9).

**Figure 2:**
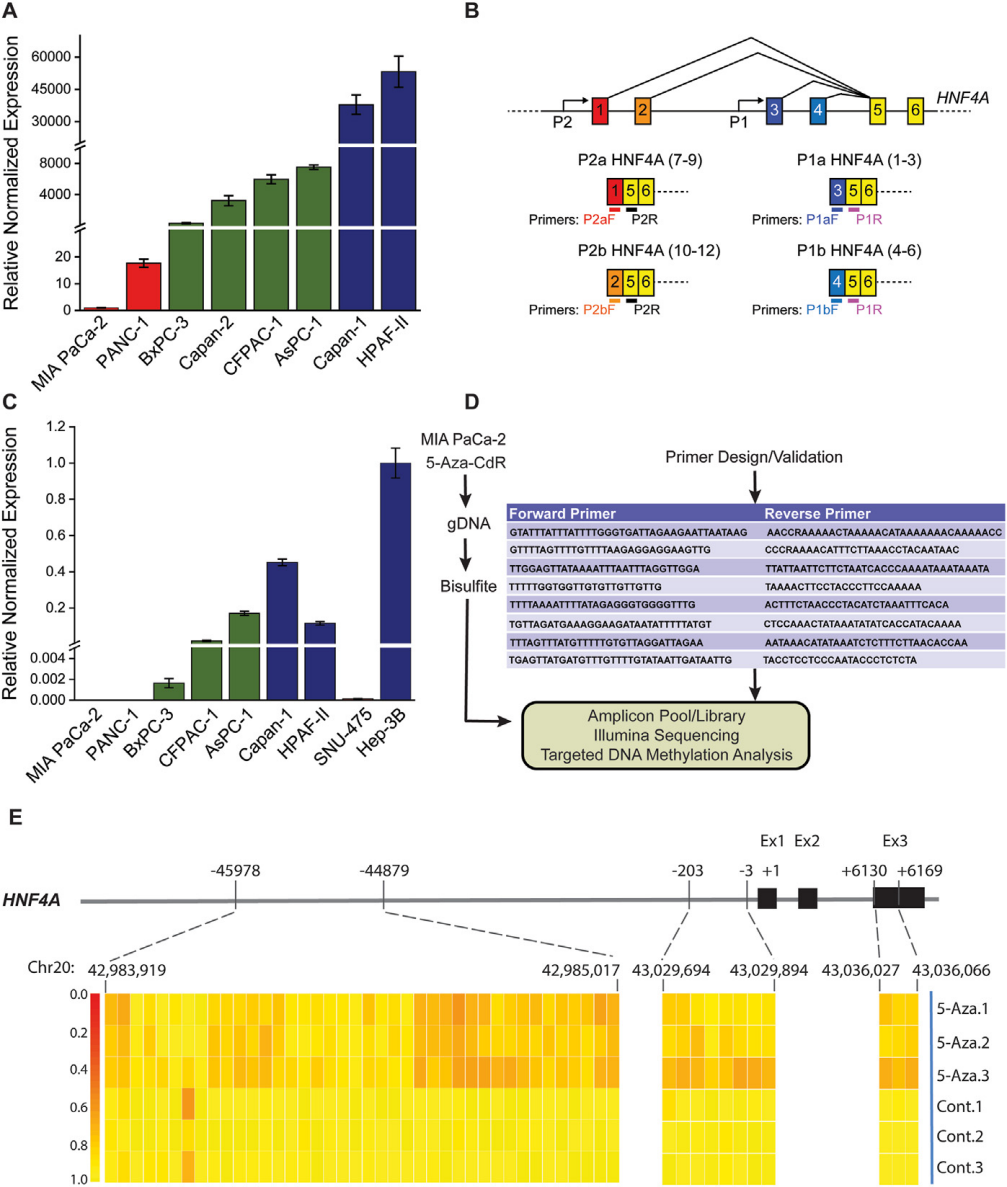
Verification of HNF4A CpG methylation sites through bisulfite sequencing. (A) HNF4A expression as assessed by RT-qPCR, in 8 pancreatic cancer cell lines. Expression was normalized to GAPDH and β-actin levels and results were expressed as mean ± SEM compared to the low HNF4A expressing cell line, MIA PaCa-2 (set as 1). (B) Diagram illustrating the generation of HNF4A isoforms through transcriptional regelation by P1 and P2 promoters and alternative splicing. Different primers were designed to recognize the 4 subgroups of the 12 HNF4A isoforms. (C) HNF4A P1a isoforms expression in pancreatic and liver (SNU-475 and Hep-3B) cancer cell lines as assessed by RT-qPCR. Expression was normalized to GAPDH and β-actin levels and results were expressed as mean ± SEM compared to the high HNF4A expressing cell line, Hep-3B (set as 1). (D) Diagram illustrating the experimental design for the evaluation of HNF4A methylation through bisulfite sequencing. (E) Heatmaps of the methylation ratio across the HNF4A locus, at the single CpG site level, for untreated (Cont) or 5-AZA-CdR–treated MIA PaCa-2 cells (5-Aza). Cells were treated with 5-Aza (1μM), for 48 h. Analysis was performed in 40 CpG sites spanning from a distal locus upstream of +1 position to exon 3.

To validate the HNF4A methylation microarray data, we performed targeted bisulfite sequencing in pancreatic cancer cells. We first checked the HNF4A mRNA levels, by means of RT-qPCR, to determine low-HNF4A–expressing cell lines (Figure 2A). It is now well documented that HNF4A expression is regulated by 2 promoters P1 and P2 of the HNF4A locus. In combination with alternative splicing, they produce 12 different isoforms.^23^ It has been proposed that the expression of HNF4A isoforms depends on the activation of the 2 promoters in a tissue-specific manner. To address their expression in pancreatic cancer cell lines, we designed primers specific for the 4 subgroups of isoforms: P1a, P1b, P2a, and P2b (Figure 2B and Table A1). For comparison, we have included one high and one low-HNF4A–expressing liver cancer cell lines. Our data show that all isoforms are represented and co-ordinately suppressed in low HNF4A in pancreatic cancer cell lines (Figure 2C and Figures A10–A12). Furthermore, RT-pPCR analysis of normal and cancer pancreatic tissues showed that both P1 and P2 HNF4A isoforms are suppressed in cancer (Figure A13).

The low-HNF4A–expressing cell lines (MIA PaCa-2) were treated with the demethylating agent 5-Aza-CdR (5-Aza); genomic DNA samples were subjected to bisulfite treatment and multiplex amplified using validated designed primers for HNF4A (Figure 2D and Table A2). This analysis verified the exact CpG sites of HNF4A hypermethylation: 40 CpG in a distal locus (−45,978 nt to −44,879 nt), 8 in proximal promoter (−203 nt to −3 nt), and 3 inside exon 3 (+6130 to 6169). Treatment with 5-Aza efficiently blocked methylation (Figure 2E). The above findings were validated in 2 additional low-HNF4A–expressing cell lines (Figures A14 and A15).

Overall, methylation array and targeted bisulfite sequencing data defined HNF4A as a novel target of hypermethylation and designated the pancreatic cancer-specific loci of HNF4A methylation.

### Transcriptional Activity of HNF4A Is Regulated by Site-Specific Methylation in the Proximal Promoter

**Figure 3:**
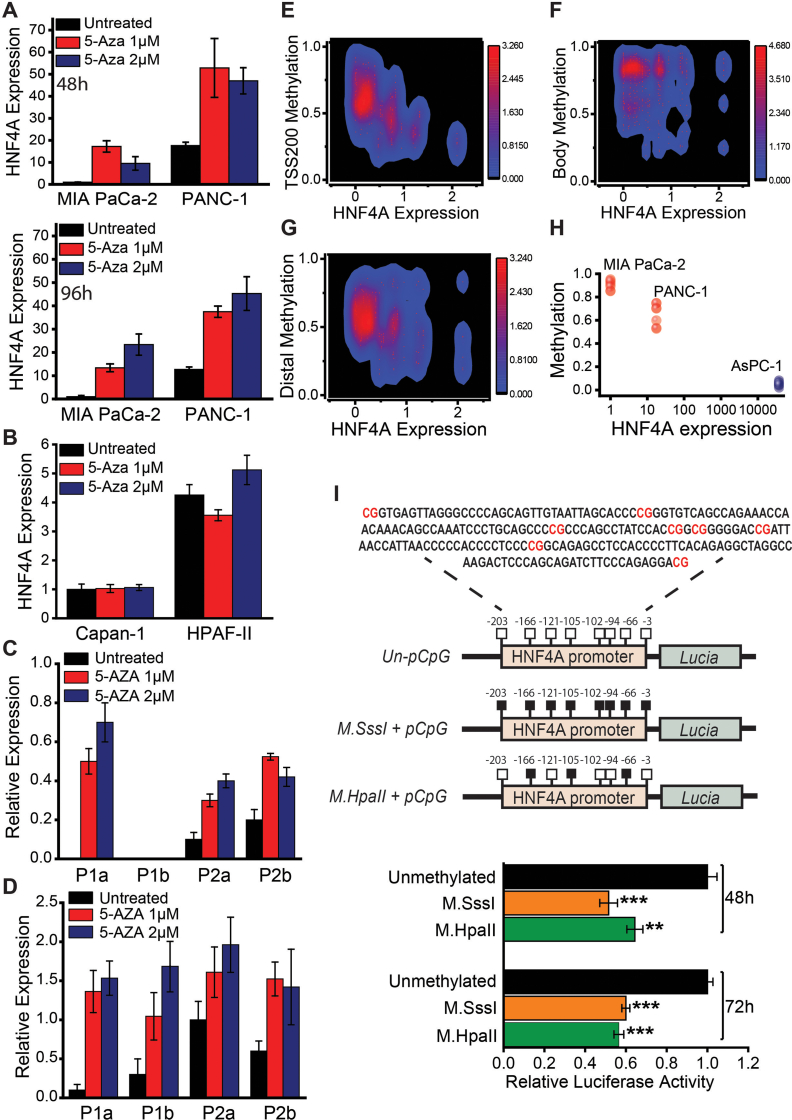
DNA methylation at the proximal promoter area regulates HNF4A transcription in pancreatic cancer. (A) HNF4A expression restoration following pancreatic cancer cell treatment with 5-AZA-CdR (5-Aza). Low HNF4A-expressing cells (MIA PaCa-2 and PANC-1) were treated with different concentrations (1 and 2 μM) for 48 or 96 h. HNF4A expression was assessed through RT-qPCR, normalized to GAPDH and β-actin levels and results were expressed as mean ± SEM compared to MIA PaCa-2 untreated cells (set as 1). (B and C) HNF4A isoforms expression in MIA PaCa-2 and BxPC-3, respectively. Cells were treated with different concentrations (1 and 2 μM) of 5-Aza, for 48 h. RT-qPCR data were normalized to GAPDH and β-actin levels. (D) HNF4A expression in high (Capan-1 and HPAF-II) HNF4A-expressing pancreatic cancer cell lines. Cells were treated with different concentrations (1 and 2 μM) of 5-Aza, for 48 h. HNF4A expression was assessed through RT-qPCR, normalized to GAPDH and β-actin levels and results were expressed as mean ± SEM compared to untreated cells (set as 1). (E–G) Pearson’s correlation analyses between HNF4A site-specific DNA methylation and gene expression, in human pancreatic cancer tissues. Correlations in TSS200 (proximal promoter area, from −200 nt to −3 nt), distal promoter, and the gene body. (H) Pearson’s correlation analyses between HNF4A site-specific DNA methylation (TSS200 area) and gene expression, in pancreatic cancer cell lines. DNA methylation is expressed in normalized beta values and gene expression assessed by RT-qPCR is expressed in comparison to MIA PaCa-2 cells (set as 1). (I) Methylation of the *HNF4A* promoter area using the pCpGfree-basic-*Lucia* reporter plasmid. *Upper panel*: Diagram illustrating the HNF4A cloned promoter fragment containing 8 CpG sites, highlighted in red on the sequence (represented as lollipops). *M.SssI* and *M.HpaII* enzymes were used to methylate the HNF4A promoter region. *Lower panel*: HEK293T cells were transiently transfected with unmethylated, M.SssI or M.HpaII methylated reporter constructs and luciferase activity was measured in cell supernatants at 48 and 72 h after transfection. The cells were co-transfected with pCMV-Cypridina Luc Vector which secretes a variant of Cypridina luciferase and was used for normalization. Results were expressed as mean ± SEM of *Lucia*/*Cypridina* activity compared to the unmethylated (Un) respective control (set as 1). Asterisks denote statistically significant differences, ∗∗∗*P* < .001, Student’s *t*-test.

We next employed 4 different sets of experiments, to address whether DNA methylation is the mechanism underlying HNF4A decreased expression in pancreatic cancer. First, we examined the effects of DNA demethylation on HNF4A mRNA levels, following treatment with 5-Aza. Inhibition of methylation was able to restore HNF4A expression, only in the low-HNF4A–expressing cell lines (Figure 3A and B and Figure A16). Importantly, inhibition of methylation restored the expression of both P1 and P2 isoforms (Figure 3C and D). Second, we integrated the HNF4A cancer-specific methylation with expression data. We found that methylation in the proximal promoter (Figure 3E) inversely correlates to gene expression (Pearson’s correlation: −0.60 to −0.68 and *P* value: 1.2 × 10^−4^ to 2.62 × 10^−5^). On the other hand, methylation in exon and distal regions (Figure 3F and G) displayed either no or low correlation (Pearson’s correlation values: −0.04 and −0.32, respectively). Third, we followed a similar approach in established pancreatic cancer cell lines, following DNA methylation, assessed through 450K DNA methylation array, and expression analyses. In accordance with the data from tissues, methylation of the proximal promoter inversely correlates to HNF4A expression in the low-HNF4A–expressing cells (MIA PaCa-2 and PANC-1, Figure 3H). Finally, to provide a direct functional link between proximal promoter methylation and transcriptional activity, we conducted in vitro methylation reporter assays. The proximal promoter region (including the respective 8 CpG sites) was subcloned into the pCpGfree-basic reporter vector, carrying the *Lucia* luciferase reporter gene, and in vitro methylation was performed using different methyltransferases (Figure 3I, upper panel). The results revealed that total (all 8 CpG sites methylated by *M.Sss*I) or partial (only 3 CpG sites methylated by *M.Hpa*II) methylation of the proximal promoter region significantly suppressed HNF4A transcription (Figure 3I, lower panel). These experiments support the notion that DNA methylation affects HNF4A expression in pancreatic cancer and most importantly identify the specific promoter loci manifesting direct HNF4A transcriptional regulation.

### HNF4A Regulates Pancreatic Cancer in Vitro and in Vivo

**Figure 4:**
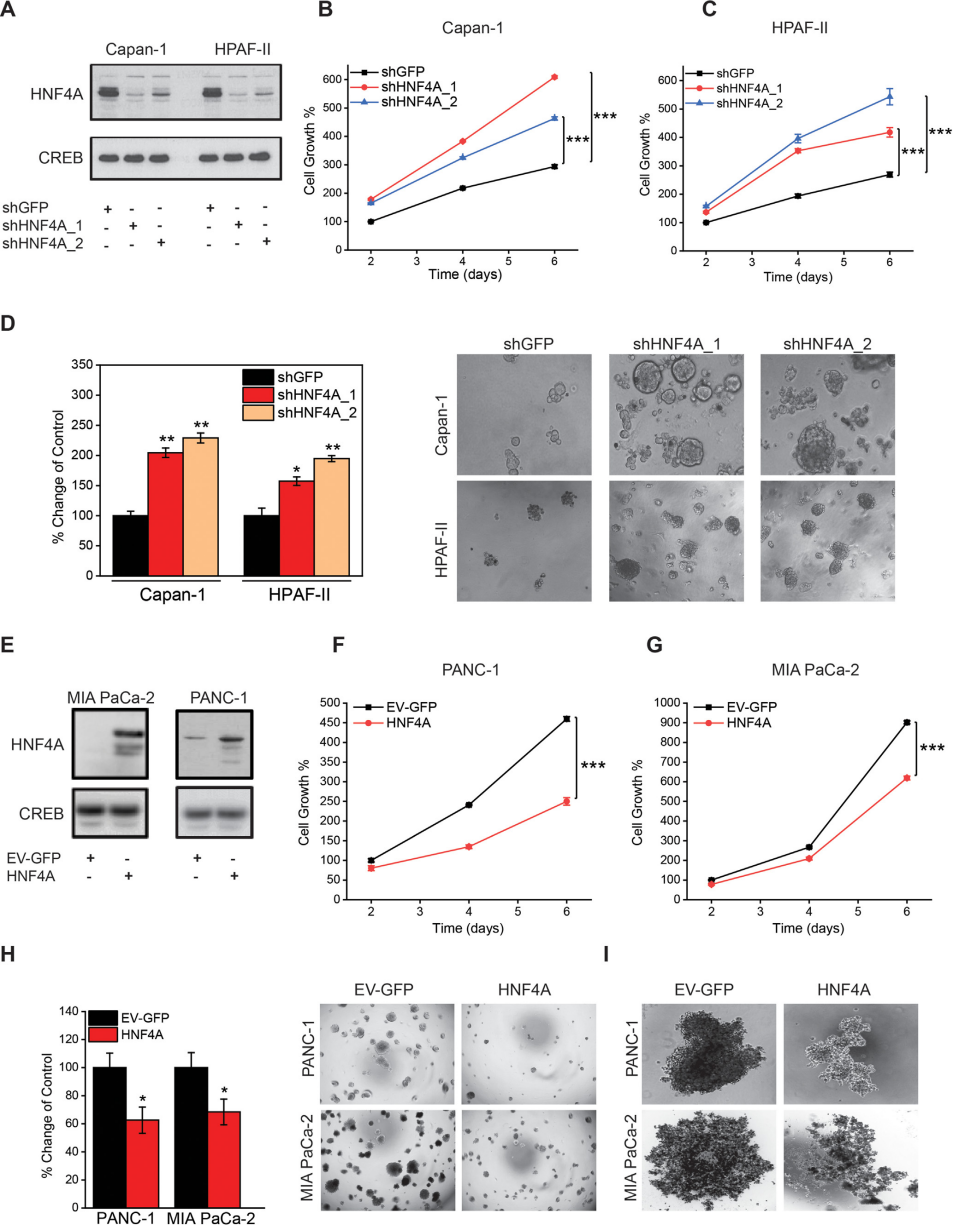
HNF4A loss-of-function and gain-of-function studies in pancreatic cancer cell lines. (A–C) Stable HNF4A knockdown was achieved by means of 2 different shRNAs (shHNF4A_1 and shHNF4A_2) in Capan-1 and HPAF-II, through lentiviral transduction. Cells transduced with shGFP were used as the control. (A) HNF4A protein levels were determined through western blot analysis and loading was assessed using an antibody against CREB. (B and C) Cell growth was assessed by the MTT and CellTiter-Glo luminescent cell viability assay. Data were expressed as mean ± SEM (the respective control cells, at day 2, were set as 100%). (D–F) HNF4A was stably overexpressed, through lentiviral transduction (HNF4A) in PANC-1 and MIA PaCa-2 cells. Cells transduced with the empty retroviral vector tagged with GFP (EV-GFP) were used as the control. (D) HNF4A protein levels were determined through western blot analysis and loading was assessed using an antibody against CREB. (E and F) Cell growth was assessed by the MTT and CellTiter-Glo luminescent cell viability assay. Data were expressed as mean ± SEM (the respective control cells, at day 2, were set as 100%). (G and H) Anchorage-independent cell growth was assessed by soft agar assays for 5 days. Data were expressed as the mean number of colonies ± SEM (respective control cells set as 100). Representative images were acquired at a 10× and 4× magnification, respectively, using an Evos microscope. (I) Spheroid formation assays using the ultra-low attachment 96-well plate method for PANC-1 (1000 cells/well) and the hanging drop method for MIA PaCa-2 (30,000 cells/20 μL drop). Representative images were acquired on day 7, at a 10× magnification, using an Evos microscope. Asterisks denote statistically significant differences, ∗*P* < .05, ∗∗*P* < .01, ∗∗∗*P* < .001, Student’s *t*-test.

To evaluate the functional role of HNF4A in pancreatic cancer, we performed a series of in vitro loss and gain of functional assays using 4 different pancreatic cancer cell lines. Transient HNF4A inhibition, by 2 different siRNAs in Capan-1 and HPAF-II cell lines, revealed that pancreatic cancer cell growth is significantly induced by HNF4A knockdown (Figures A17 and A18, Table A1). In the same line, stable HNF4A depletion (Figure 4A–C and Figure A19) significantly induced pancreatic cancer cell growth and colony formation (Figure 4D). Furthermore, matrigel-coated transwell assays revealed that HNF4A knockdown significantly induces pancreatic cancer cell invasiveness (Figure A20). On the other hand, stable overexpression of HNF4A in MIA PaCa-2 and PANC-1 cell lines (Figure 4E and Figure A21) significantly suppressed cell growth and colony formation (Figure 4F, G, and H). In accordance, HNF4A restoration significantly impaired pancreatic cancer cell invasiveness (Figure A22). Importantly, HNF4A restoration inhibited the ability of PANC-1 and MIA PaCa-2 cells to form spheroids/organoids in ultra-low attachment plates or hanging drops, respectively (Figure 4I). Additionally, to evaluate the effect of HNF4A on the stemness of the spheroids, we screened for expression of cancer stem cell markers, including CD24, CD44, NESTIN, OCT4, and SOX2. First, we observed that cancer stem cell markers expression is higher in PANC-1 HNF4A expressing spheroids when compared to the respective adherent cells. Second, but most importantly, HNF4A-expressing spheroids exhibit decreased expression of CD24, CD44, and SOX2 when compared to the respective control spheroids (Figure A23 and Table A3). Taken together, these data suggest that HNF4A is a tumour suppressor in pancreatic cancer and its loss induces carcinogenic cellular properties including increased growth, colony formation, and invasiveness.

**Figure 5:**
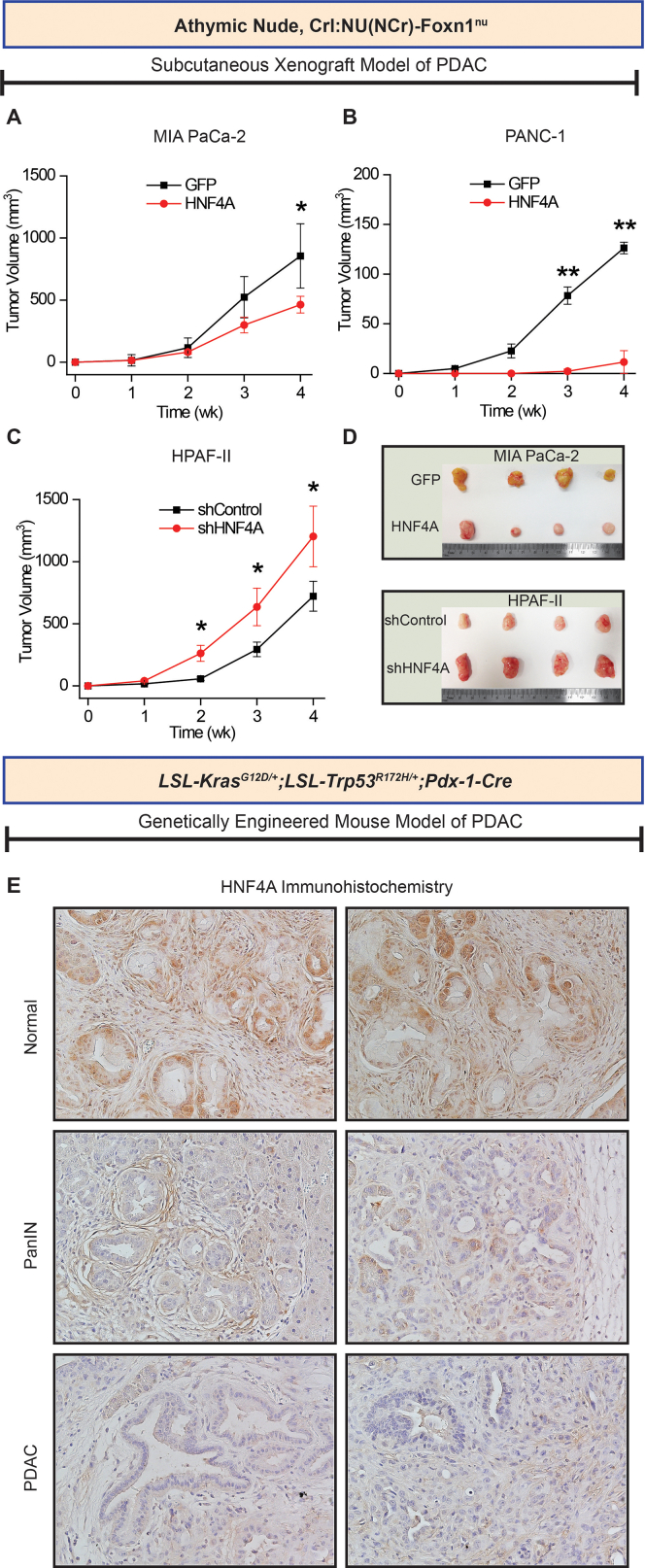
HNF4A loss is an early event and promotes PDAC growth in vivo. (A–C) Effect of HNF4A on in vivo xenograft tumour growth. HNF4A was stably overexpressed, through lentiviral transduction (HNF4A) in MIA PaCa-2 and PANC-1 cells. Cells transduced with the empty retroviral vector tagged with GFP (GFP) were used as the control. Stable HNF4A knockdown was achieved by means of shRNA in HPAF-II cells, through lentiviral transduction and cells transduced with shControl were used as the control. Cells were injected subcutaneously in NOD-SCID mice and tumor growth was monitored for a total period of 4 weeks. Tumor volumes were calculated by the equation V (mm^3^) = a × b^2^/2, where a is the largest diameter and b is the perpendicular diameter. (D) Representative images of tumours extracted from mice at the end of the experiment. (E) HNF4A is suppressed at early stages of pancreatic cancer growth in the KPC (*LSL-Kras*^*G12D/+*^;*LSL-Trp53*^*R172H/+*^;*Pdx-1-Cre*) mouse model. Tissues extracted from different stages of pancreatic cancer development were subjected to HNF4A immunohistochemical analysis (brown, HNF4A; blue, haematoxylin). PanIN, pancreatic intraepithelial neoplasia; PDAC, pancreatic ductal adenocarcinoma. For PANC-1, N = 3 mice/group. For MIA PaCa-2 and HPAF-II, N = 8 mice/group. Asterisks denote statistically significant differences, ∗*P* < .05, ∗∗*P* < .01, Student’s *t*-test.

Building on our in vitro findings, we examined the HNF4A functional role in xenograft mouse models of pancreatic cancer. Nude mice were inoculated subcutaneously with MIA PaCa-2 cells engineered to stably overexpress HNF4A, and comparisons were made to mice injected with cells transduced with the empty lentiviral vector (tagged with GFP). Our results show that HNF4A significantly suppressed tumour growth, at 4 weeks post cell engraftment (Figure 5A). Similarly, HNF4A overexpression abrogated the tumorigenic capability of PANC-1 cells, when compared to the respective control (Figure 5B). Interestingly, mice inoculated with PANC-1 GFP cells developed palpable tumours 1 week post cell engraftment, whereas in the ones inoculated with PANC-1 HNF4A, the same number of cells produce tumours 3 weeks post cell engraftment. In another setting, nude mice were inoculated subcutaneously with HPAF-II cells stably depleted of HNF4A or the empty lentiviral vector. Accordingly, HNF4A loss significantly induced tumour growth from the second week after cell inoculation (Figure 5C and D).

To further validate the in vivo significance, we assessed HNF4A expression at different stages of murine pancreatic cancer development. Hence, we have used the KPC (*Pdx1-Cre*; *LSL-KrasG12D*; *Trp53R172H*) genetically engineered mouse model of pancreatic cancer. Pancreatic tissues were collected at different stages of the disease and HNF4A was detected through immunohistochemistry (Figure 5E). HNF4A expression declines from the early stages of the disease, as assessed in intraepithelial neoplasia lesions (PanIN), and exhibits the minimum staining in later stages PDAC has developed.

Overall, these data demonstrate that HNF4A loss is an early event during pancreatic cancer development, significantly contributing to increased growth and aggressiveness.

### HNF4A Loss Is an Early Event in Human Pancreatic Cancer and Correlates With Poor Patient Survival

Having shown HNF4A loss in one cohort of pancreatic cancer patients (Stanford, USA) and one commercially available TMA, we sought to discern whether this event has any clinical significance. To this end, we examined HNF4A expression in 2 extra cohorts of patients and performed correlation analyses with clinicopathological parameters including tumor stage and survival.

**Figure 6:**
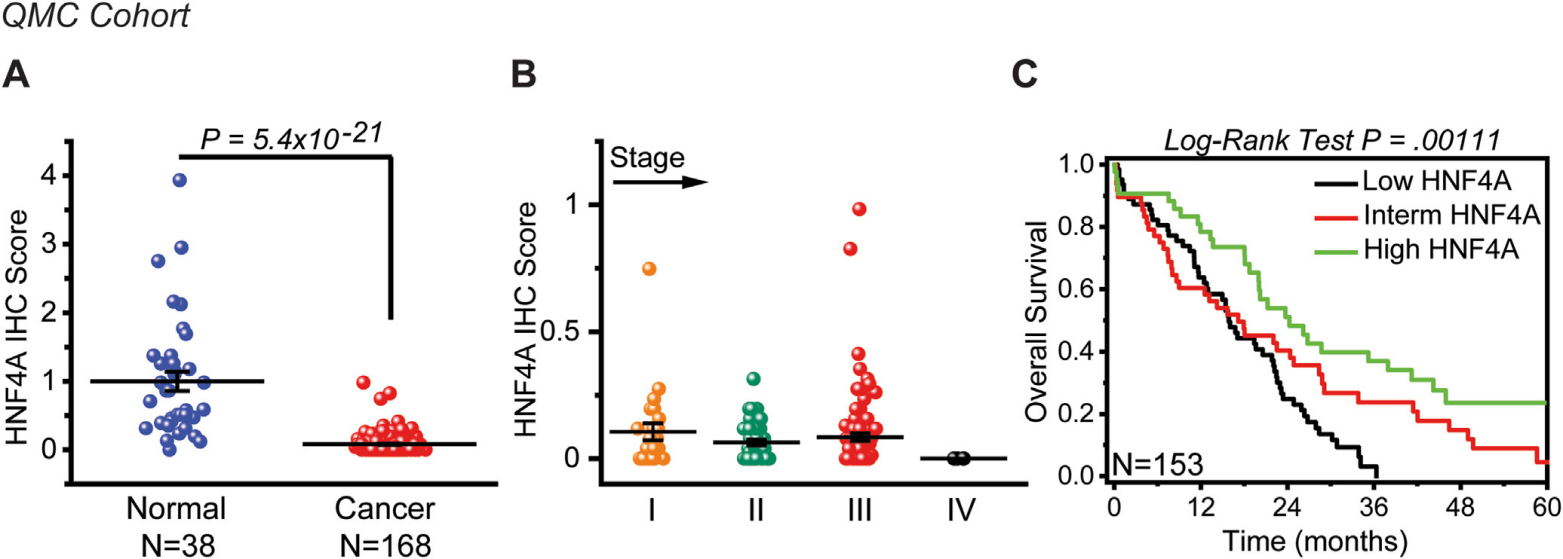
Discovery cohort of 168 pancreatic cancer patients reveals that HNF4A loss is an early event and correlates with poor overall survival. HNF4A expression was assessed by immunohistochemical analysis, in 168 pancreatic cancer and 38 normal (uninvolved) tissues (QMC: Queen’s Medical Centre, Nottingham, UK). (A) Staining and scoring of tissues was performed in Histopathology Department of QMC and results were expressed as mean ± SEM compared to normal tissues (set as 1). (B) Assessment of HNF4A staining in 168 pancreatic cancer tissues according to their tumor stage. Results were expressed as mean ± SEM compared to normal tissues (set as 1). (C) Survival analysis in 153 patients divided into low, intermediate (interm), and high HNF4A expression subgroups. Survival estimates were generated using the Kaplan-Meier method and compared using log-rank tests.

We have analysed HNF4A expression, through immunohistochemistry, in a discovery cohort of 168 pancreatic cancer and 38 normal tissues (QMC cohort, UK). Scoring of the immunostained tissues revealed a statistically significant suppression of HNF4A in human pancreatic cancer, when compared to normal (Figure 6A). In accordance with our data from the KPC animal model, HNF4A expression is significantly suppressed from stage I indicating that HNF4A loss is an early event in human pancreatic cancer (Figure 6B). Important to note is that HNF4A levels did not exhibit any significant changes across stages. To further elucidate the clinical significance of HNF4A in pancreatic cancer, we correlated its expression with patient overall survival. Kaplan-Meier curves and Cox’s proportional hazard modelling were developed. Interestingly, Kaplan-Meier survival analysis (Figure 6C) revealed that low HNF4A expression correlates with poor survival (N = 153, *P* = .00111). Clinicopathological analysis revealed that HNF4A staining is associated with tumour stage and not with a variety of other clinicopathologic variables, such as gender, tumour size, and age (Table 1). Most importantly, univariate and multivariate analysis of the survival data with the Cox model indicated an increased risk of death for patients with low HNF4A expression (Table 1).

**Table 1:** Effect of HNF4A Expression on Overall Survival was Assessed in 153 Patients (QMC Cohort), Using the Univariate and Multivariate Cox Proportional Hazard Analyses

Clinicopathological Parameters	Case (%)	Univariate Cox		Multivariate Cox
		HR (95% CI)	*P* value	HR (95% CI)
Age (mean ± SD: 64.17 ± 10.55)				
>60	49 (32.0%)			
≤60	104 (68.0%)	1.085 (0.746–1.578)	.669	1.379 (0.943–2.015)
Sex				
Female	63 (41.2%)			
Male	90 (58.8%)	1.248 (0.891–1.850)	.179	1.379 (0.943–2.015)
TNM staging				
I	21 (13.73%)			
II	36 (23.53%)	2.966 (1.434–6.136)	.00337	2.748 (1.320–5.719)
III	92 (60.13%)	3.300 (1.699–6.411)	.00042	3.285 (1.674–6.444)
IV	4 (2.61%)	16.490 (4.915–55.331)	5.68e-06	14.297 (4.127–49.527)
Tumour size (cm)				
>3	82 (53.59%)			
≤3	71 (46.41%)	1.044 (0.7286–1.496)	.815	1.125 (0.769–1.645)
HNF4A score (high/low)				
High	50 (32.68%)			
Low	103 (67.31%)	1.627 (1.09–2.428)	.0173	1.379 (0.919–2.066)

Low HNF4A expression (highlighted in bold) is significantly associated to a Hazard Ratio (HR) >1. HR of 1 indicates no difference between 2 groups of patients, while HR >1 indicates an increased risk of death/failure for the group listed.

CI, confidence interval; HNF4A, hepatocyte nuclear factor 4A; HR, hazard ratio; SD, standard deviation.

**Figure 7:**
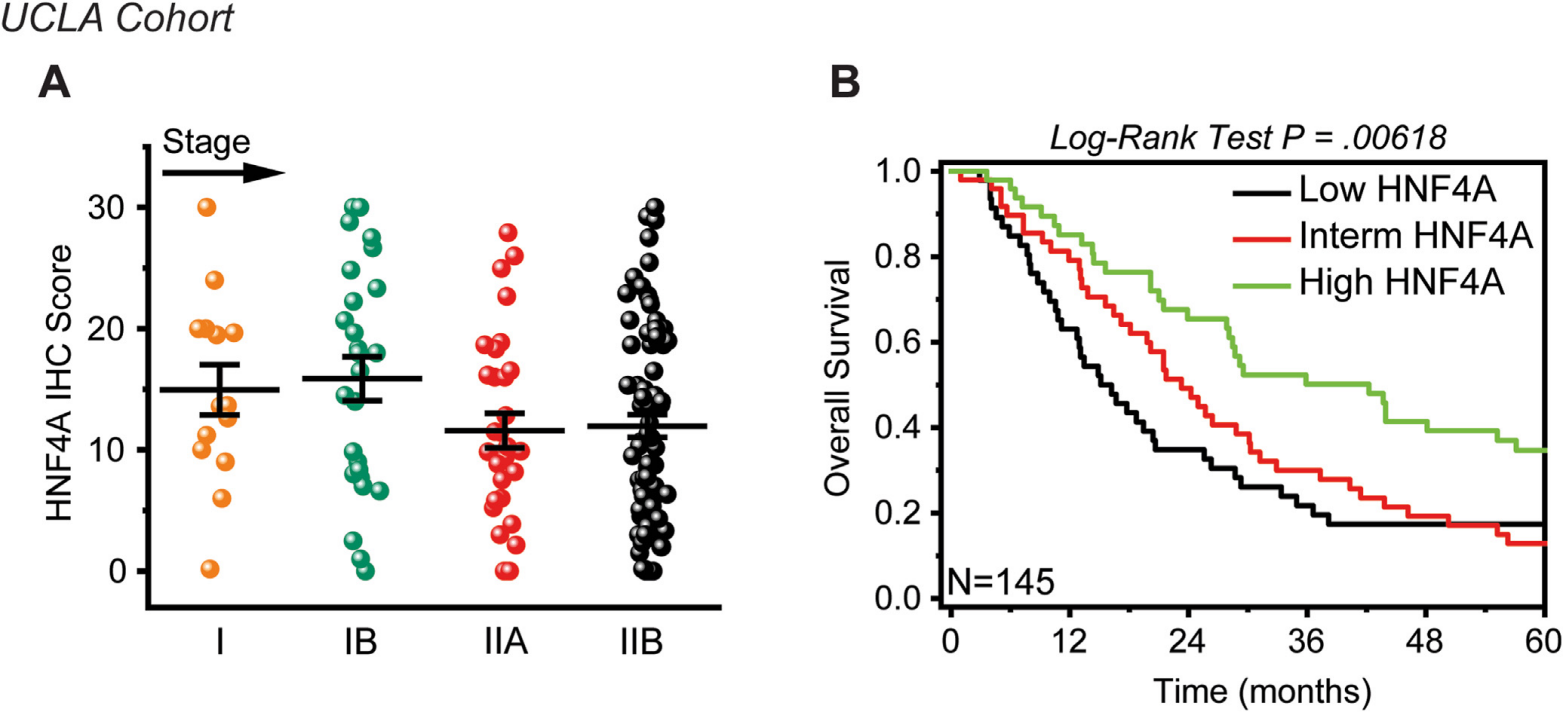
HNF4A loss indicates an increased risk of death in a validation cohort of 145 pancreatic cancer patients. HNF4A expression was assessed by immunohistochemical analysis, in 145 pancreatic cancer tissues (UCLA, USA). (A) Staining and scoring of the HNF4A immunostained tissues was performed in the Department of Pathology at UCLA Medical Center. Assessment of HNF4A staining in pancreatic cancer tissues according to their tumor stage. (B) Survival analysis in patients divided into low, intermediate (interm), and high HNF4A expression subgroups. Survival estimates were generated using the Kaplan-Meier method and compared using log-rank tests.

To increase the robustness of our findings, we conducted immunohistochemistry for HNF4A in a validation cohort (UCLA cohort, USA) of 145 tissue specimens from pancreatic cancer patients. In line with the above data, HNF4A loss is an early event (Figure 7A), with no significant changes across stages, and low HNF4A expression correlates with poor survival (Figure 7B) as indicated by Kaplan-Meier analysis (N = 145, *P* = .00618). Most importantly, univariate and multivariate Cox regression analysis revealed a statistically significant increased risk of death (hazard ratio = 1.9131 with *P* value = .002 and hazard ratio = 1.808 with *P* value = .006, respectively) for patients with low HNF4A expression (Table 2).

**Table 2:** Effect of HNF4A Expression on Overall Survival Was Assessed in 145 Patients (UCLA Cohort), Using the Univariate and Multivariate Cox Proportional Hazard Modelling

Clinicopathological Parameters	Case (%)	Univariate Cox		Multivariate Cox
		HR (95% CI)	*P* value	HR (95% CI)
Age (mean ± SD: 60.30 ± 11.33)				
>60	49 (33.79%)			
≤60	96 (66.21%)	0.958 (0.646–1.421)	.8325	0.937 (0.629–1.395)
Sex				
Female	70 (48.28%)			
Male	75 (51.72%)	0.742 (0.508–1.082)	.1212	0.745 (0.505–1.098)
TNM staging				
I	40 (27.59%)			
II	105 (72.41%)	1.787 (1.142–2.795)	.01101	1.719 (1.077–2.744)
Tumour size (cm)				
>3	87 (60%)			
≤3	58 (40%)	1.243 (0.848–1.819)	.2641	0.929 (0.617–1.398)
HNF4A score (high/low)				
High	49 (33.79%)			
Low	96 (66.21%)	1.913 (1.253–2.920)	.00265	1.808 (1.177–2.776)

Multivariate Cox proportional hazards models were used to test statistical independence and significance of multiple predictors with backward selection performed using the Akaike Information Criterion. Low HNF4A expression (highlighted in bold) is significantly associated to a Hazard Ratio (HR) >1. HR of 1 indicates no difference between 2 groups of patients, while HR >1 indicates an increased risk of death/failure for the group listed.

CI, confidence interval; HNF4A, hepatocyte nuclear factor 4A; HR, hazard ratio; SD, standard deviation.

Taken together, our data strongly suggest that HNF4A expression declines from the early stages of human pancreatic cancer; its suppression is sustained during pancreatic cancer progression and confers to poor patient survival.

## Discussion

Several studies have addressed PDAC plasticity based on a plethora of factors including genetic, environmental, and the tumour microenvironment. Nevertheless, the PDAC transcriptional heterogeneity and the different levels of epigenetic regulation that might explain the phenotypic plasticity are unknown to a large extent. In this study, DNA methylation analysis revealed that in pancreatic cancer, thousands of genes exhibit an altered methylation profile, when compared to normal pancreata.

Pathway analysis for the specific gene set revealed a novel differentially methylated network, consisting of HNF family transcriptional regulators adding a new dimension in HNFs cross-regulation and exhibit synergistic relationship in human disease.^24^ Our study is the first one that identifies HNF1A as a pancreatic cancer-specific hypermethylated gene. In agreement with our data, HNF1A loss has been correlated with poor patient survival and increased chemoresistance.^25^ HNF4A hypermethylation in PDAC comes in line with previous reports^8^^,^^26^ in which HNF4A methylation was correlated to suppression of gene expression. Interestingly, it has been proposed recently that HNF1A suppression coincides with HNF4A loss in PDAC.^27^ It is well established that HNF4A and HNF1A belong to a unique transcriptional circuit; however, tissue and cell specificity seem to dictate bidirectional regulation with epistatic role.^28^^,^^29^

Analysis of the HNF4A locus revealed cancer-specific hypermethylation in the promoter and gene body regions. To test whether HNF4A locus susceptibility to methylation is translated into alterations in gene expression, we evaluated HNF4A levels in a significant number of human tissues. Using 3 independent cohorts of patients, we found a significant suppression of HNF4A in pancreatic cancer. In normal pancreas, HNF4A was predominantly detected in the nuclear compartment of ductal epithelium and the surrounding acinar tissue. HNF4A expression and cellular/subcellular localization in pancreatic cancer has attracted attention; however, the results reported are conflicting. In databases, amplification of the HNF4A locus^30^ and upregulation of HNF4A mRNA^31^^,^^32^ are reported for human PDAC. In human protein atlas,^33^ HNF4A is detected in well-differentiated epithelial structures of PDAC and localized in the cytoplasm or not expressed in poorly differentiated PDAC. In another report, HNF4A was absent from the human normal pancreatic ducts, strongly expressed in the nucleus of PanIN-2 and PanIN-3 lesions and barely or not detectable in PDAC.^34^ Transcriptomic analyses in bulk PDAC tumours revealed HNF4A loss, mainly in the squamous and pancreatic progenitor subtypes.^8^^,^^33^

Herein, we provide several lines of evidence indicating that DNA methylation is responsible for HNF4A loss in pancreatic cancer, and most importantly, we identify the specific promoter loci responsible for direct HNF4A transcriptional regulation. First, inhibition of methylation induced restoration of HNF4A expression in the low-HNF4A–expressing cell lines. Second, targeted bisulfite sequencing analyses revealed the exact CpG sites of HNF4A hypermethylation. Third, integration of the HNF4A cancer-specific methylation with expression data, both in tissues and cell lines, demonstrated that the methylation status of the proximal promoter region inversely correlates to gene expression. Finally, and most importantly, in vitro methylation reporter assays provided the direct functional link between specific promoter cytosine-guanine site methylation and transcriptional activity. It is documented that P1 promoter-regulated HNF4A isoforms are predominantly expressed in liver, whereas P2 promoter-regulated HNF4A isoforms are expressed in the normal pancreas. However, evidence supports the expression of specific P1 isoforms in normal pancreas and pancreatic cancer cell lines.^23^^,^^35^ Our data show that at least some P1 HNF4A isoforms are expressed at comparable levels in pancreatic cancer cell lines and tissues. In fact, DNA demethylation in low-HNF4A–expressing cells restored the expression of P1 isoforms and increased the expression of P2 isoforms. The methylation of the distal promoter may also contribute to the suppression of HNF4A isoforms, while the methylation of the proximal promoter may also regulate the P2 isoforms as part of the gene body. This hypothesis could be addressed by CRISPR/CAS9 methylation approaches targeting the proximal and distal HNF4A promoters.

Epigenetic silencing of HNF4A in PDAC has been recently attributed to alterations of H3K4me3 and H3K27ac patterns around the TSS.^33^ Consistent with this theory, we have identified significant promoter hypomethylation of histone demethylase KDM2B in pancreatic cancer tissues. KDM2B preferentially demethylates H3K4me3 and H3K36me2,^36^ promotes pancreatic cancer,^37^ and interacts with HNF4A.^38^ Taken together, different levels of transcriptional and epigenetic regulation might determine HNF4A expression in pancreatic cancer. All these events could act in a synergistic and/or sequential way to silence HNF4A during PDAC progression.

Apart from recent studies focusing on HNF4A dysregulation, its functional role in pancreatic cancer is far from clear. Herein, we show that HNF4A behaves as a tumour suppressor regulating cellular properties such as cell growth, colony and spheroid formation, and invasiveness. The tumour suppressive role was further verified in vivo, in a xenograft mouse model of pancreatic cancer. HNF4A loss significantly induced, while HNF4A overexpression suppressed tumour growth.

A recent report based on a pancreatic cancer mouse model proposed that HNF4A loss is necessary for the phenotypic switch from a classical to a basal subtype.^24^ Here, we demonstrate that HNF4A expression during murine pancreatic cancer development in the KPC (*Pdx1-Cre*; *LSL-KrasG12D*; *Trp53R172H*) genetically engineered mouse model progressively declines from the early stages of the disease from PanIN lesions to PDAC. Similarly, in hepatocellular carcinoma, we have previously shown that HNF4A loss is an early event that drives hepatocellular transformation, and this suppression becomes a stable event through an epigenetic switch.^39^ Furthermore, our data come in line with a recent study, proposing that HNF4A loss drives metabolic reprogramming at an early stage of PDAC progression inducing glycolysis and WNT pathway activation.^26^

In accordance with our data from the KPC animal model, immunohistochemical staining in 2 cohorts of patients revealed that HNF4A loss is an early event, significantly suppressed at stage I, with nonsignificant changes across stages. This observation comes in line with the notion that abnormal methylation-associated silencing of tumour suppressor genes precedes genetic mutations and may drive tumour initiation.^40^ To further elucidate the clinical significance of HNF4A in pancreatic cancer, we correlated its expression with patient survival. This is the first study that correlates HNF4A expression with patient survival in PDAC and most importantly the expression is assessed following scoring of immunostained tissues and not relying on bulk tumor profiling. Kaplan-Meier curves and Cox’s proportional hazard modelling, in both patient cohorts, indicate that low HNF4A expression correlates with poor patient survival.

Taken together, our data strongly suggest that HNF4A expression declines from the early stages of human pancreatic cancer; its suppression is sustained during pancreatic cancer progression and confers to poor patient survival. Although HNF4A has been considered an orphan nuclear receptor, and the nature and effect of its ligand on transcriptional activity are not clear, it should be highlighted that its ligand-binding pocket is not constitutively occupied by fatty acids. The exploration of the HNF4A interactome, its post-translational modifications, or the development of ligand-mimicking small molecules aiming at residual HNF4A activation may prove a viable approach in pancreatic cancer therapeutics.
