# Supplementary material for: Promoter Methylation Leads to Hepatocyte Nuclear Factor 4A Loss and Pancreatic Cancer Aggressiveness
Source: Gastro Hep Adv. 2024 Apr 24;3(5):687–702. doi: 10.1016/j.gastha.2024.04.005 (PMC11330932; doi:10.1016/j.gastha.2024.04.005)
Supplement: Supplementary Figures and Tables [file mmc1.docx]

**APPENDIX**

**SUPPLEMENTARY METHODS**

*Human Methylation 450K Array*

For global methylation profiling, we used the Illumina Infinium HumanMethylation450 (HM450) BeadChip (Illumina), which interrogates DNA methylation status of > 450,000 CpGs and >99% of all genes. Genomic DNA was isolated using the DNeasy Blood & Tissue Kit (69504, Qiagen). Bisulfite conversion was performed on 1 µg of genomic DNA, using the EZ-96 DNA Methylation Kit (Zymo Research) according to the manufacturer’s instructions. Bisulfite-converted DNA was whole genome amplified (WGA) and enzymatically fragmented prior to hybridization to BeadChip arrays. The oligomer probe designs of HM450 arrays follow the Infinium I and II chemistries, in which locus-specific base extension follows hybridization to a methylation-specific oligomer.

The level of DNA methylation at each CpG locus was scored as beta (β) value calculated as (M/(M+U)), ranging from 0 to 1, with 0 indicating no DNA methylation and 1 indicating fully methylated DNA. Data were background corrected, quantile normalized and extracted using of Illumina Genome Studio Methylation Module. Of the 485,577 CpG probes on the array, we filtered out probes with high detection p values, probes with a SNP within 10 base pairs of the target CpG and repeat regions and probes on X and Y chromosomes, leaving 371,478 probes. The term ‘hyper-methylation’ was used when there was an increased DNA methylation in patients compared to controls and, the term ‘hypo-methylation’ was used when we observed a decreased DNA methylation in patients compared to controls.

Wilcoxon rank-sum tests were used to compare methylation array data between pancreatic cancer patients and healthy controls. Magnitude of DNA methylation changes was assessed using methylation beta values. Correction for multiple comparisons was performed using FDR (Benjamini-Hochberg) approach. DNA methylation β-values for the selected probes on pancreatic cancer patients and control subjects were represented graphically by plotting heatmaps, generated using the R package ‘heatmap.plus’ (R Development Core Team. R: A language and environment for statistical computing. Vienna, Austria: 2010). DNA methylation data were complemented by transcriptomic analyses. A corrected *P* value, denoted as, ‘q’ ≤ .05 was considered significant. Genes associated with hyper-methylation of CpG sites as well as down-regulation of its expression were considered as epigenetically silenced and visualized using starburst plot.

*Immunohistochemistry*

For DAB immunostaining, FFPE sections were deparaffinized with xylene (3x5 min) followed by treatment with serial dilutions of ethanol (100%, 100%, 95% and 95%, 10 min each) and two changes of ddH2O. Antigen unmasking was achieved by boiling the slides (95-99°C) for 10 min, in 10mM sodium citrate. Sections were rinsed three times with ddH2O, immersed in 3% H2O2 for 20 min, washed twice with ddH2O, once with TBS-T (TBS, 0.1% Tween-20) and blocked for 1h with 5% normal goat serum (5425) in TBS-T. HNF4A antibody (3113) was diluted 1:200 in Signal Stain antibody diluent (8112) and incubated with the sections overnight at 4°C. Sections were washed three times with TBS-T, incubated for 1h at room temperature with SignalStain Boost (HRP-Rabbit, 8114, Cell Signaling Technology), washed three times with TBS-T, and stained with the DAB Peroxidase Substrate Kit (SK-4100, Vector Laboratories) for 30min. Tissues were counterstained with hematoxylin QS (H-3404, Vector Laboratories), and images were captured with a Nikon 90i Upright Microscope equipped with a Nikon Digital Sight DS-Fi1 colour camera, using the NISElements image acquisition software.

*Immunoblot Analysis*

Cells were homogenized using RIPA buffer (9806, Cell Signaling Technology), followed by sonication, and total cell extracts were separated by SDS-PAGE followed by transfer onto PVDF membranes under standard procedures. HNF4A immunoblot analysis was achieved using a monoclonal HNF4A antibody (3113, Cell Signaling Technology). CREB analysis was employed to assess total protein loading using a CREB monoclonal antibody (9104, Cell Signaling Technology).

*Cell growth assay*

Cells were plated in quadruplicates in 96-well plate (1x103 cells/well) and cell growth was assessed 2, 4 and 6 days later. Cell growth was assessed using two different assays: the CellTiter Glo Luminescence Cell Viability Assay (G7571, Promega) or 3-[4,5-dimethylthiazol-2-yl]-2,5-diphenyltetrazolium bromide thiazolyl blue (MTT) assay (M6494, Life Technologies). Data were expressed as mean fluorescence (arbitrary units) ± SEM or as mean O.D. (at 490nm) ± SEM (control cells at day 2 were set as 100%), respectively.

*Anchorage-independent cell growth assay*

Triplicate samples from each cell line and treatment were assayed in 96-well plates for colony formation using the CytoSelect Cell Transformation kit (CBA-130, Cell Biolabs). The number of colonies was quantified after 5 days by counting the entire area of each well divided in four fields, using a grid and an Evos microscope at a 10X magnification. Data were expressed as the mean percentage change ± SEM of colonies in the respective control cells (set as 100).

*Invasion assay*

Invasion through matrigel has been conducted by using BD BioCoat Matrigel invasion chambers (354480, BD Biosciences), according to manufacturer’s instructions. Briefly, FBS-containing media were used as the chemoattractant, non-invading cells on the top side of the membrane were removed and invading cells were fixed and stained with 0.1% crystal violet, 24h post seeding. The cells that migrated through the filter were quantified by counting the entire area of each filter divided in four fields, using a grid and an Evos microscope at a 20X magnification. Data were expressed as the mean percentage change ± SEM of control invading cells (set as 100).

**SUPPLEMENTARY FIGURES**


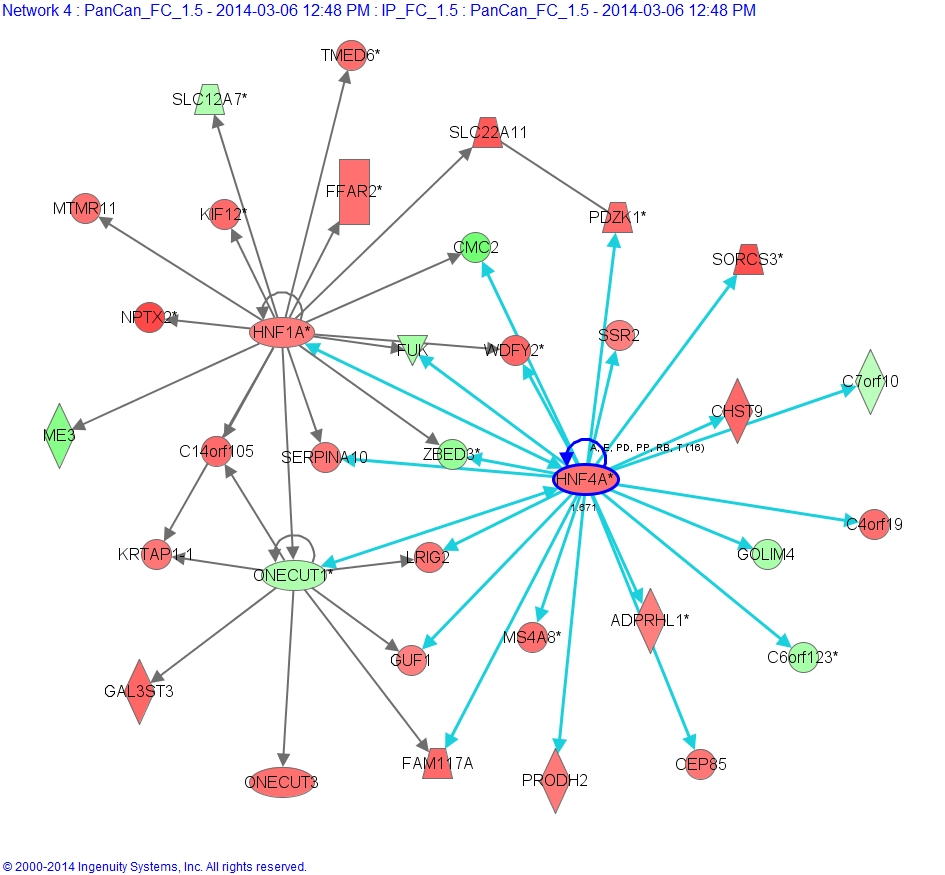


**Figure A1:** Ingenuity pathway analysis of genes associated with a robust differential promoter methylation revealed hepatocyte Nuclear Factor (HNF) family network as the top differentially methylated.

**
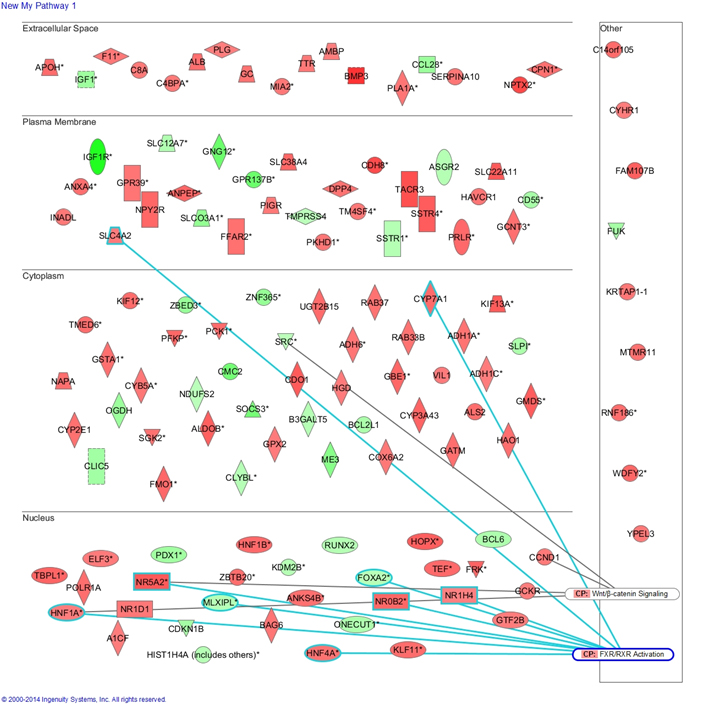
**

**Figure A2:** An HNF family network was identified, following Ingenuity Pathway Analysis of the genes corresponding to the 7,241 differentially methylated promoter sites (*P* < .05, FC ≥ 1.5).

**
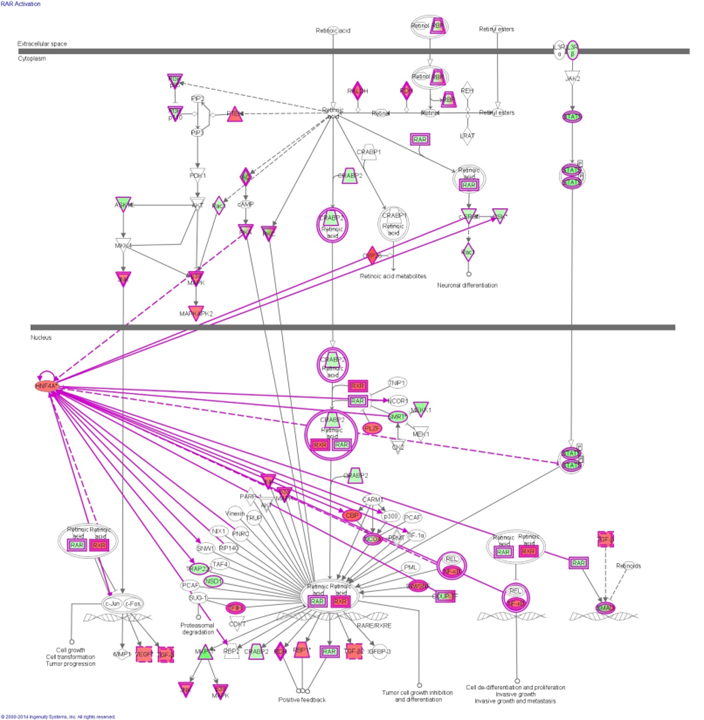
**

**Figure A3:** HNF4A was identified, following Ingenuity Pathway Analysis of the genes corresponding to the 7,241 differentially methylated promoter sites (*P* < .05, FC ≥ 1.5).

**
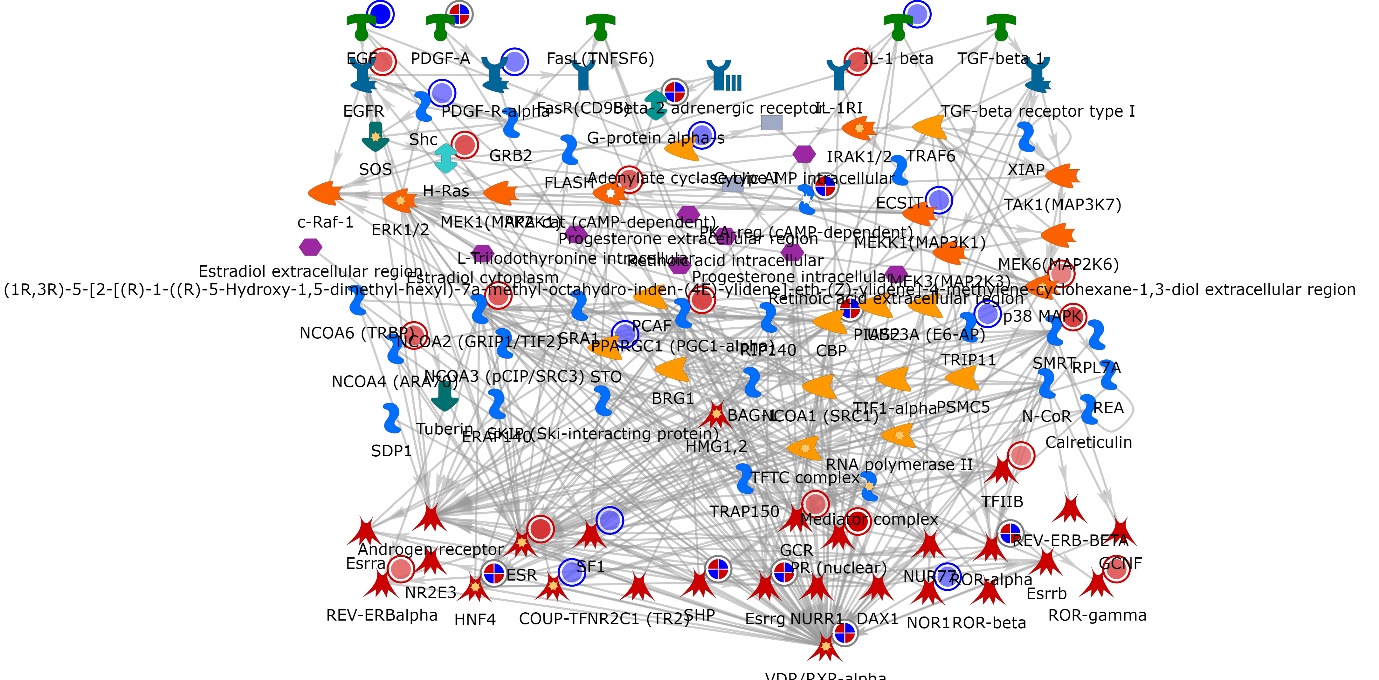
**

**Figure A4:** HNF4A was identified following Metacore Pathway Analysis of the genes corresponding to the 7,241 differentially methylated promoter sites (*P* < .05, FC ≥ 1.5).


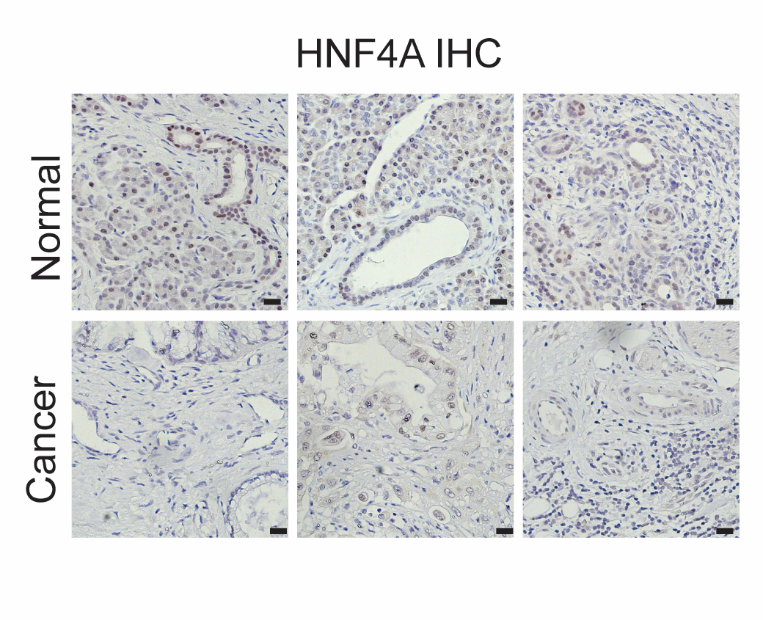


**Figure A5:** Immunohistochemical analysis for HNF4A in control and pancreatic cancer tissues (brown, HNF4A; blue, haematoxylin). Scale bar, 20µm.

**
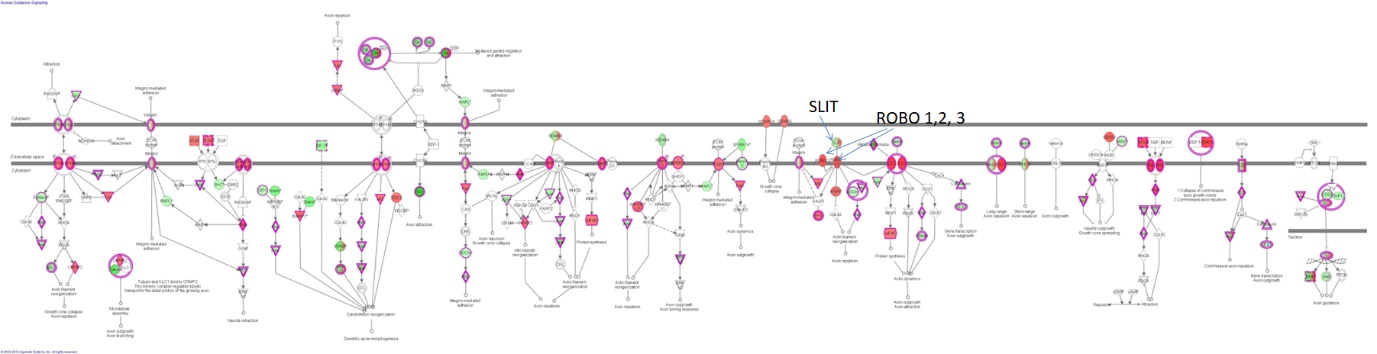
**

**Figure A6:** Axon guidance signalling pathway was identified following Ingenuity Pathway Analysis of the genes corresponding to the 7,241 differentially methylated promoter sites (*P*< .05, FC ≥ 1.5).


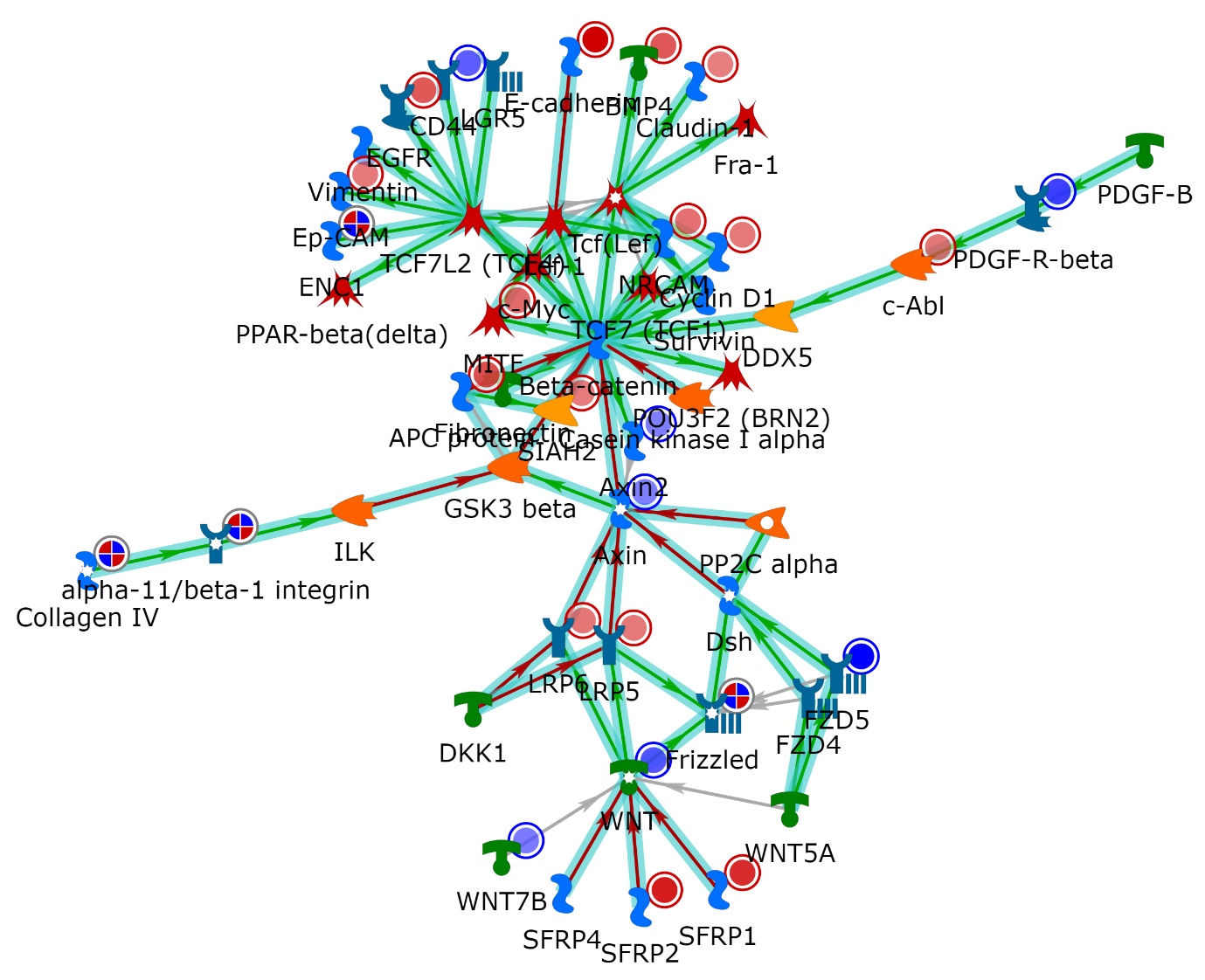


**Figure A7:** WNT/beta-catenin signalling pathway was identified following Metacore Pathway Analysis of the genes corresponding to the 7,241 differentially methylated promoter sites (*P* < .05, FC ≥ 1.5).

**
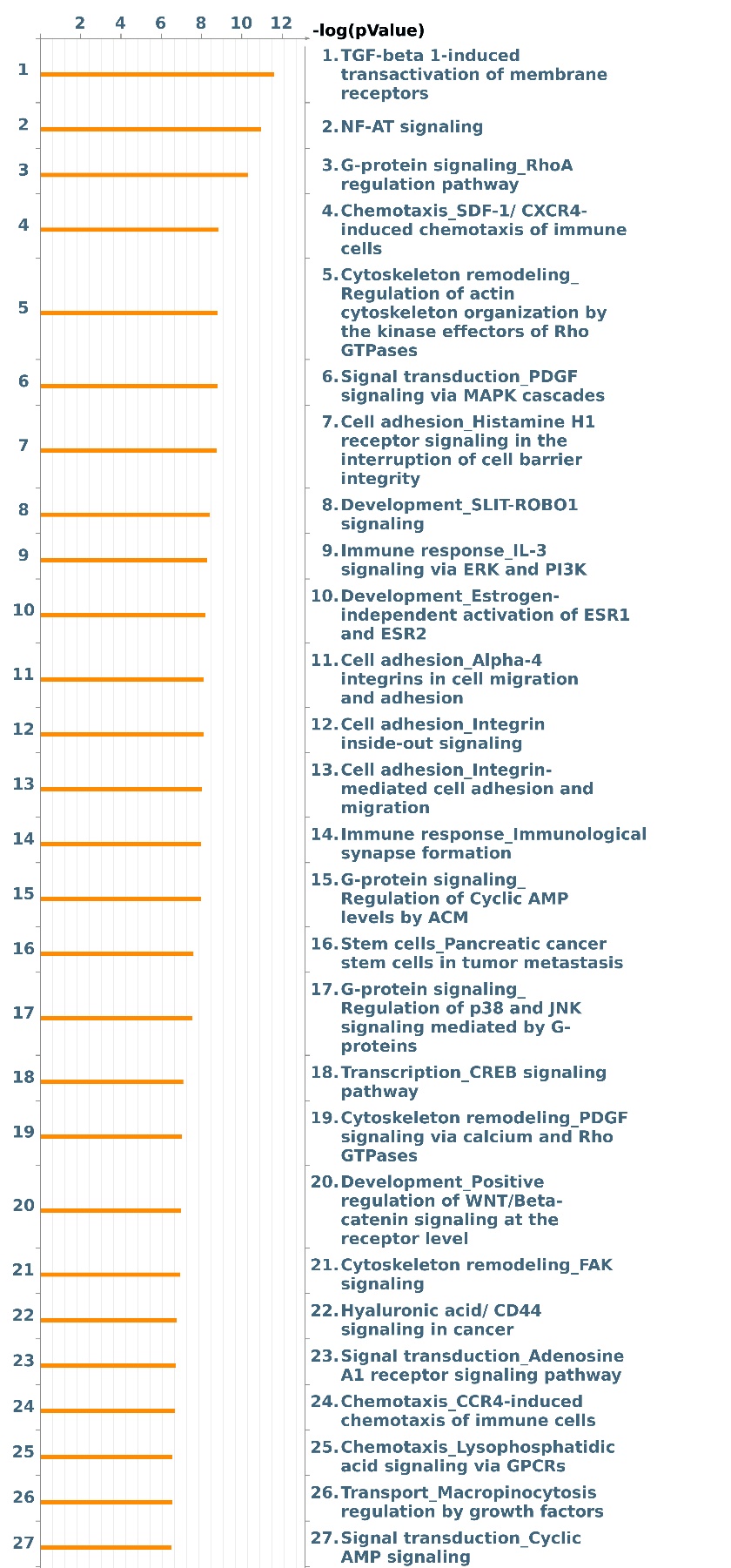
**

**Figure A8:** Top pathways, including TGF-beta and cell adhesion/integrin signalling, were identified following Metacore Pathway Analysis of the genes corresponding to the 7,241 differentially methylated promoter sites (*P* < .05, FC ≥ 1.5).

| **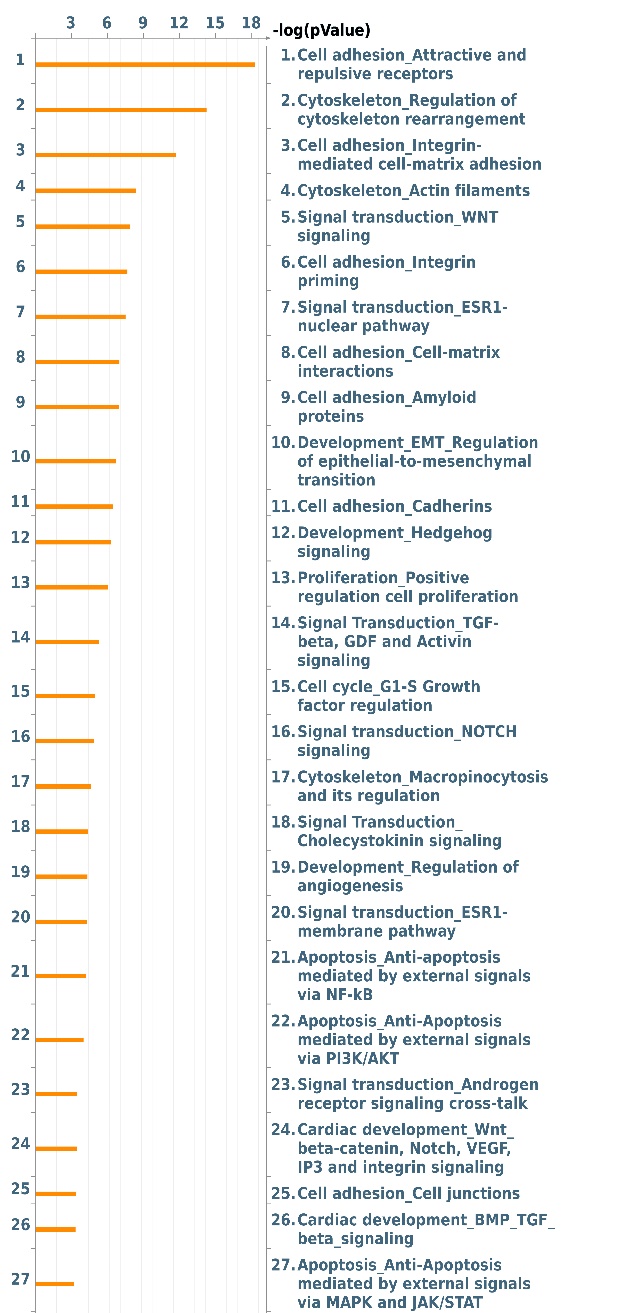** | **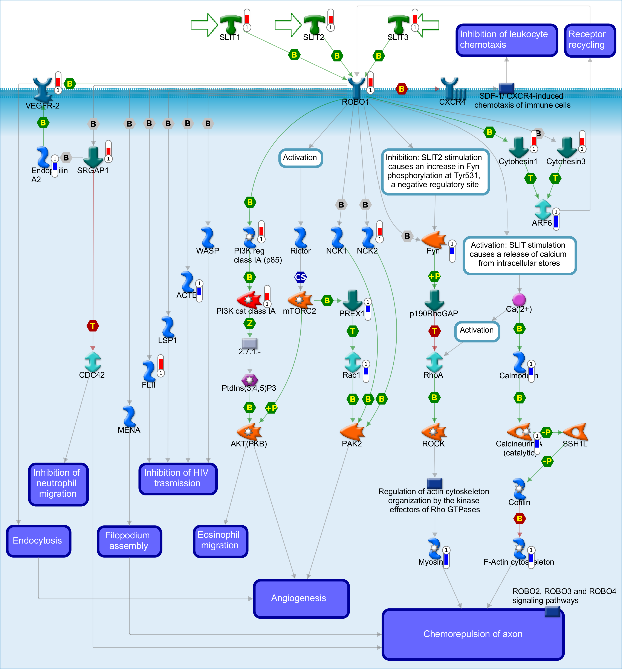** |
| --- | --- |

**Figure A9:** PI3K/AKT pathway was identified following Metacore Pathway Analysis of the genes corresponding to the 7,241 differentially methylated promoter sites (*P* < .05, FC ≥ 1.5).

**
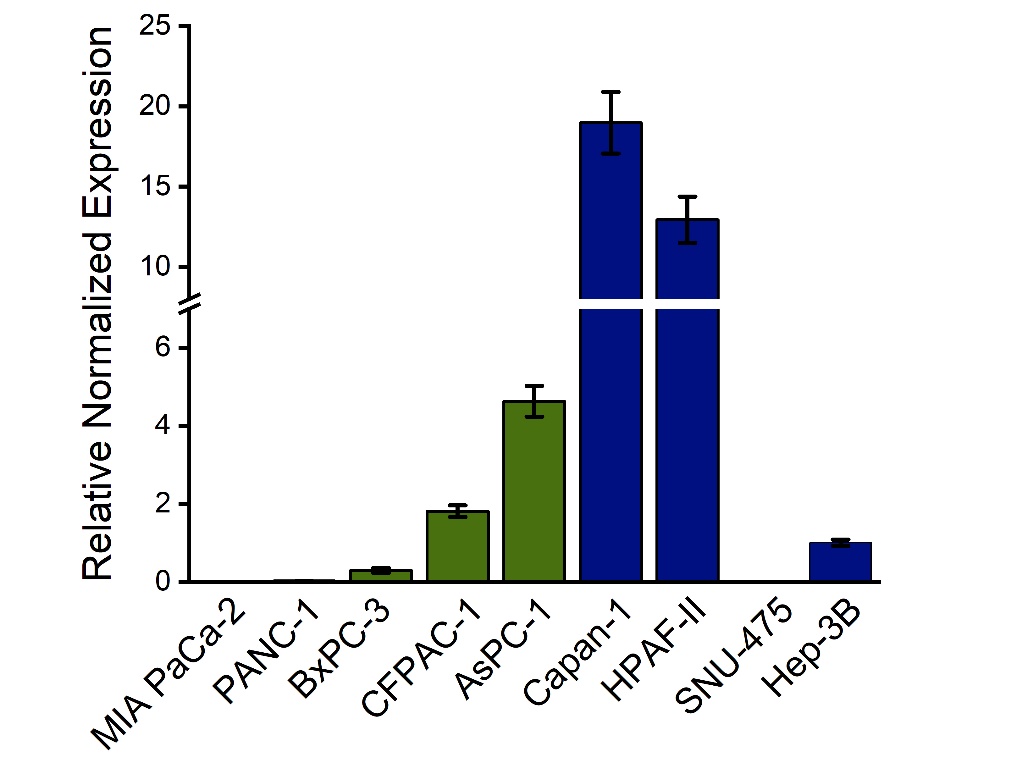
**

**Figure A10:** HNF4A P1b isoforms expression in pancreatic and liver (SNU-475 and Hep-3B) cancer cell lines as assessed by RT-qPCR. Expression was normalized to GAPDH and β-actin levels and results were expressed as mean ± SEM compared to Hep-3B (set as 1).

**
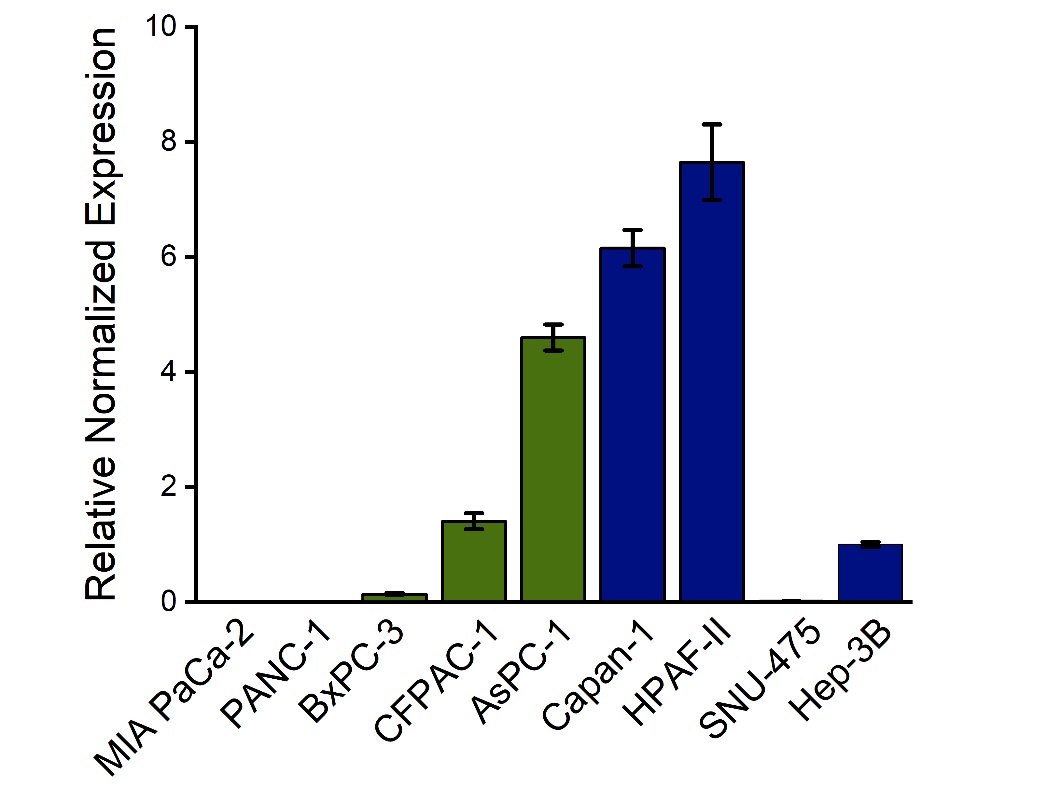
**

**Figure A11:** HNF4A P2a isoforms expression in pancreatic and liver (SNU-475 and Hep-3B) cancer cell lines as assessed by RT-qPCR. Expression was normalized to GAPDH and β-actin levels and results were expressed as mean ± SEM compared to Hep-3B (set as 1).

**
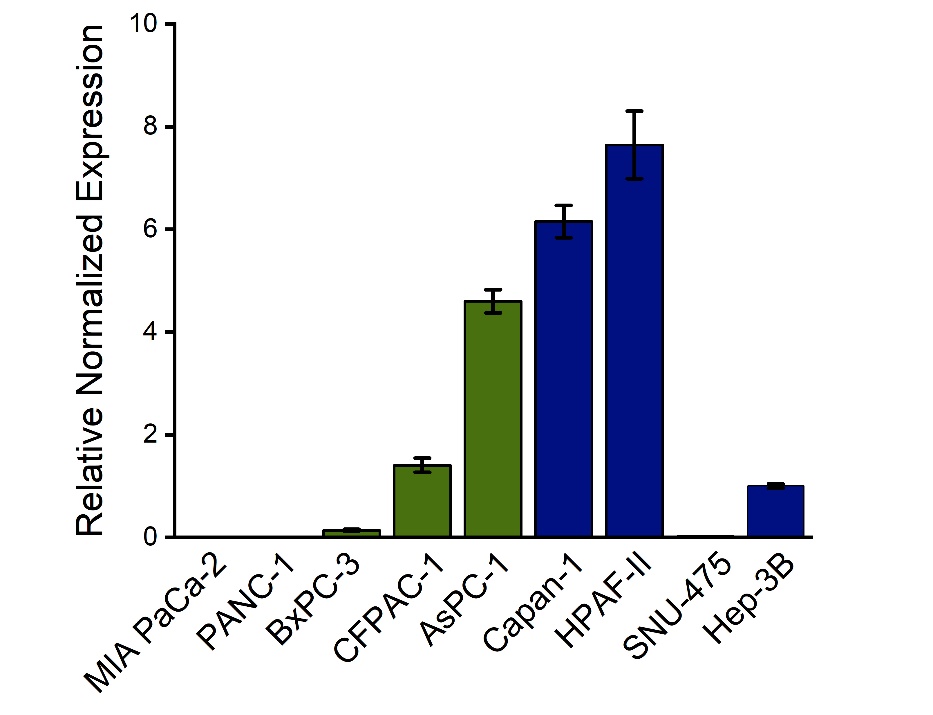
**

**Figure A12:** HNF4A P2b isoforms expression in pancreatic and liver (SNU-475 and Hep-3B) cancer cell lines as assessed by RT-qPCR. Expression was normalized to GAPDH and β-actin levels and results were expressed as mean ± SEM compared to Hep-3B (set as 1).

**
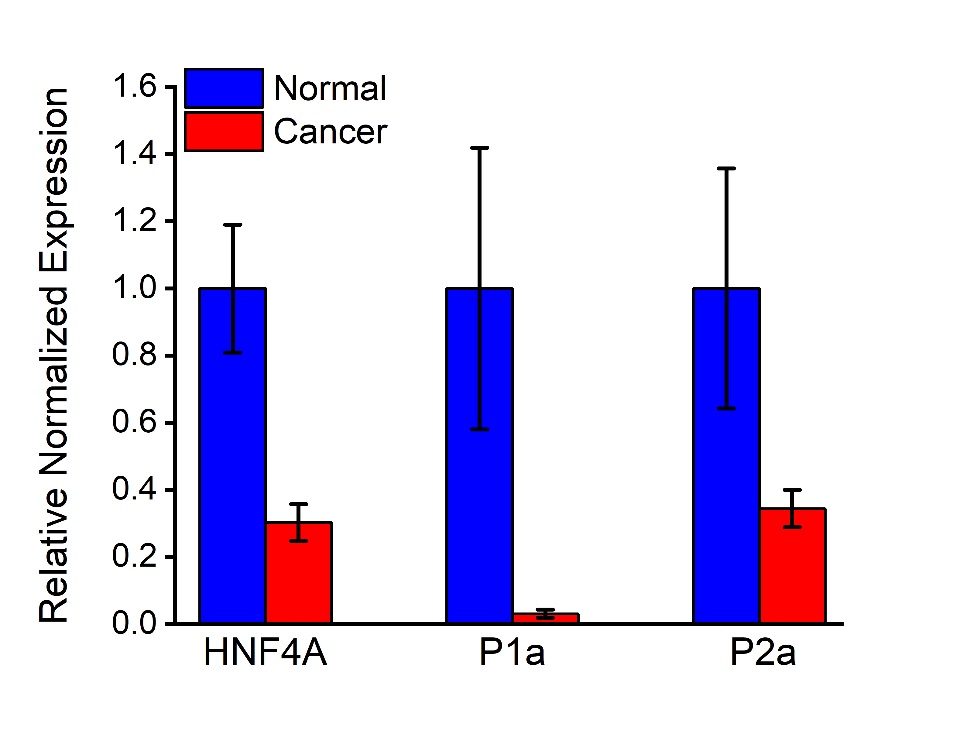
**

**Figure A13:** HNF4A, P1a and P2a isoforms expression in normal pancreas (n=5) and pancreatic cancer (n=10, as assessed by RT-qPCR. Expression was normalized to GAPDH and β-actin levels and results were expressed as mean ± SEM compared to normal tissues (set as 1).

**
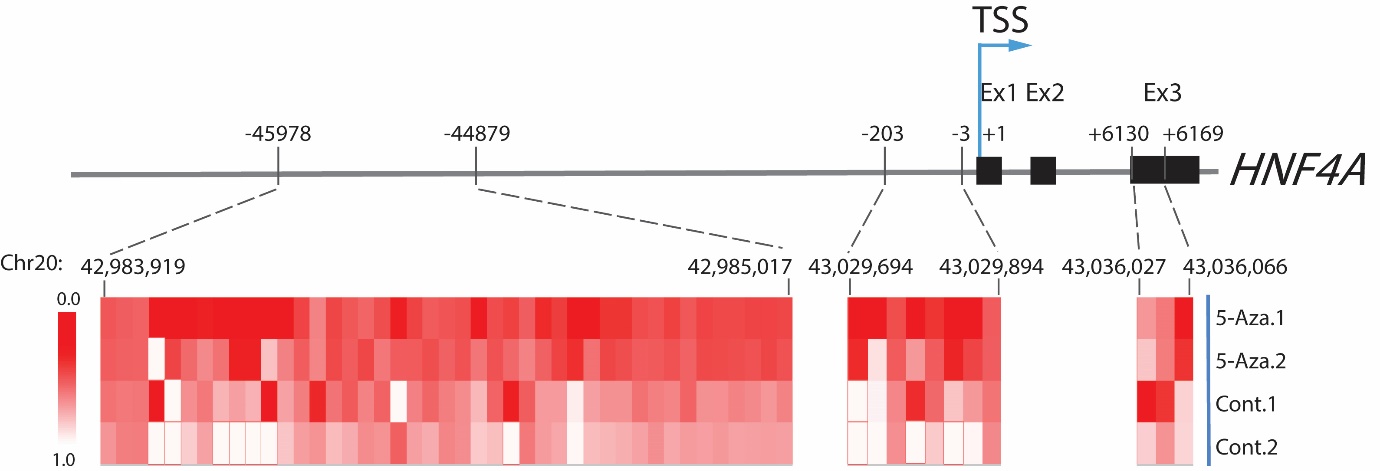
**

**Figure A14:** Verification of *HNF4A* CpG methylation sites through bisulfite sequencing. Heatmaps of the methylation ratio across the *HNF4A* locus, at the single CpG site level, for untreated (Cont) or 5-AZA-CdR-treated BxPC-3 cells (5-Aza). Cells were treated with 5-Aza (1µM), for 48 h. Analysis was performed in 40 CpG sites spanning from the distal promoter to exon 3.

**
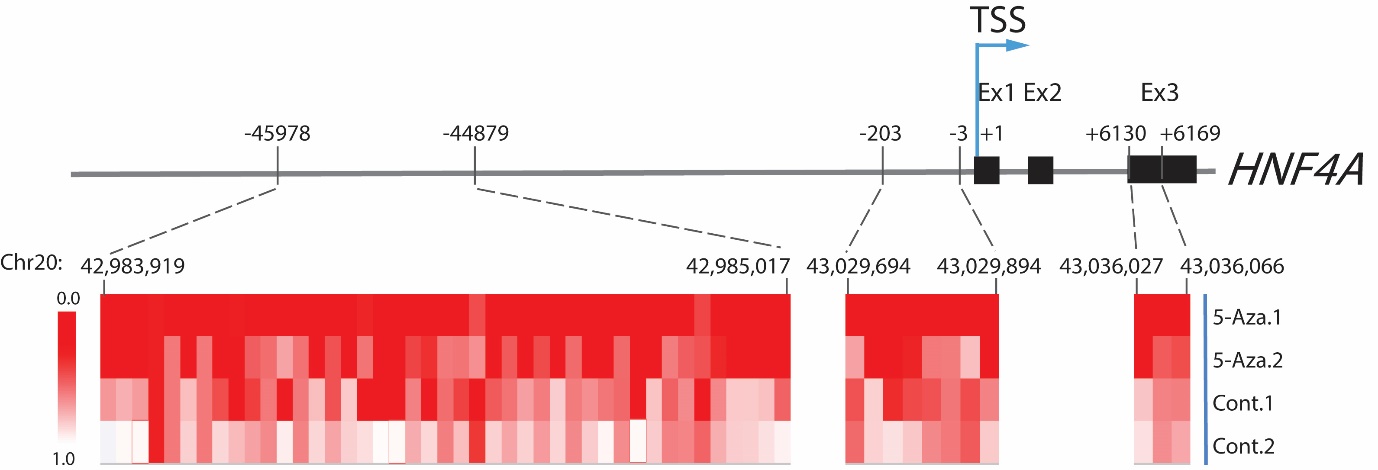
**

**Figure A15:** Verification of HNF4A CpG methylation sites through bisulfite sequencing. Heatmaps of the methylation ratio across the *HNF4A* locus, at the single CpG site level, for untreated (Cont) or 5-AZA-CdR-treated AsPC-1 cells (5-Aza). Cells were treated with 5-Aza (1µM), for 48 h. Analysis was performed in 40 CpG sites spanning from the distal promoter to exon 3.

| **A** | **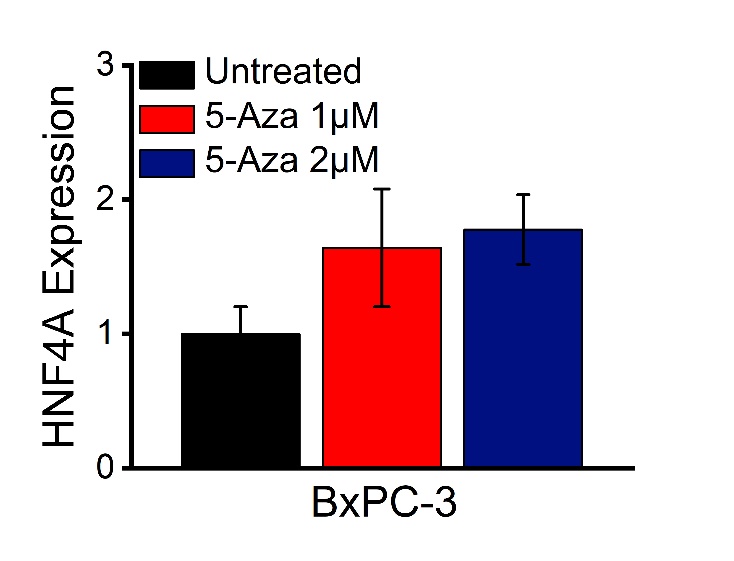** |
| --- | --- |
| **B** | **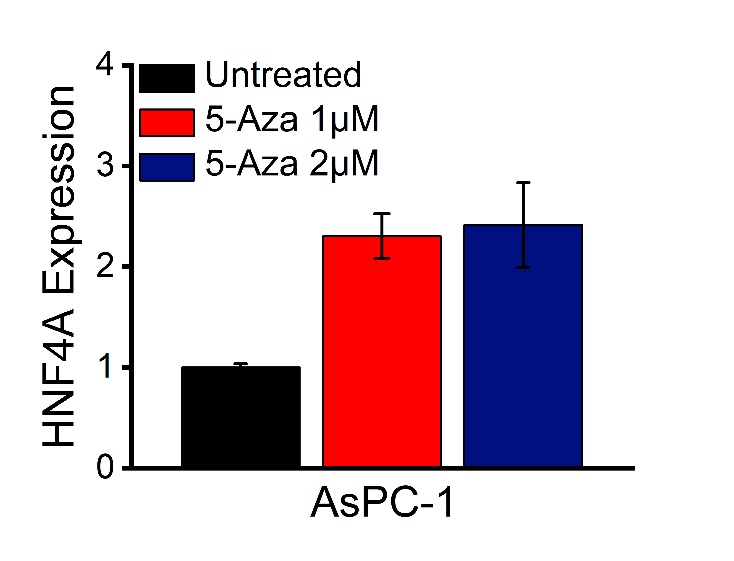** |

**Figure A16:** HNF4A expression in BxPC-3 **(A)** and AsPC-1 **(B)** pancreatic cancer cell lines. Cells were treated with two different concentrations (1 and 2 µM) of 5-AZA-CdR (5-Aza). HNF4A expression was assessed through RT-qPCR, normalized to GAPDH and β-actin levels and results were expressed as mean ± SEM compared to the respective untreated cells (set as 1).

| **A** | **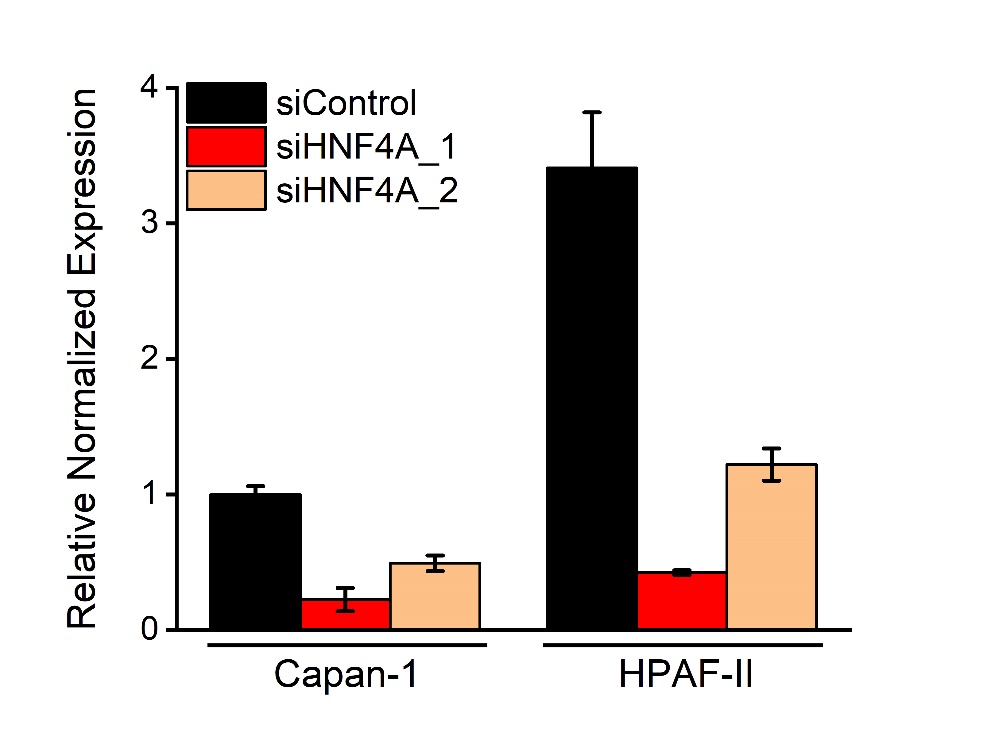** |
| --- | --- |
| **B** | **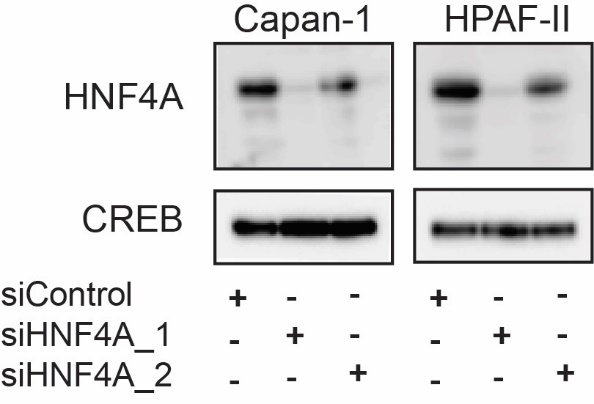** |

**Figure A17:** HNF4A knockdown efficiency by means of siRNAs. Transient HNF4A knockdown was achieved by means of two different shRNAs (siHNF4A_1 and _2) in Capan-1 and HPAF-II. Cells transfected with siControl were used as the control. **A)** HNF4A mRNA levels were assessed through RT-qPCR, normalized to GAPDH and β-actin levels and results were expressed as mean ± SEM compared to Capan-1 cells transfected with siControl (set as 1). **B)** HNF4A protein levels were determined through western blot analysis and loading was assessed using an antibody against CREB.

| **A**   |
| --- |
| **B**  **** |

**Figure A18:** HNF4A was transiently suppressed, using two different siRNAs (siHNF4A_1 and _2) in **A)** Capan-1 and **B)** HPAF-II cells. Cells transfected with siControl were used as the control. Cell growth was assessed by the CellTiter-Glo luminescent cell viability assay, 48h following the transfection. Data were expressed as mean fluorescence (arbitrary units) ± SEM (siControl cells, at day 2, were set as 100%). Asterisks denote statistically significant differences, ** *P* < .01, *** *P* < .001, Student’s t test.

| **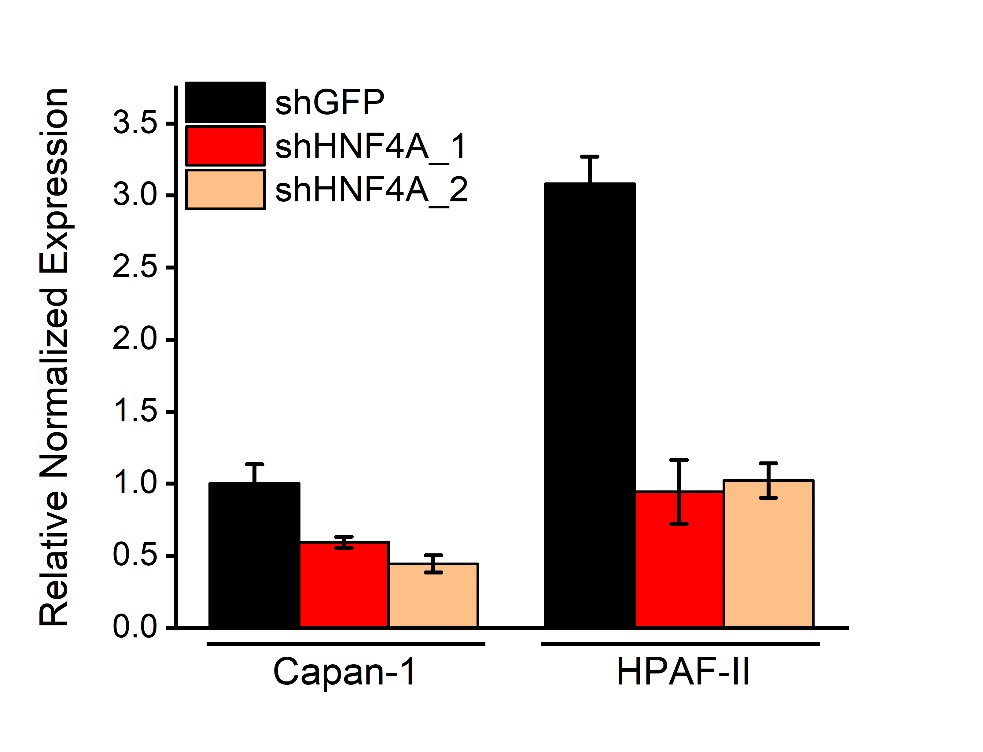** |
| --- |

**Figure A19:** Stable HNF4A knockdown was achieved by means of two different shRNAs (shHNF4A_1 and _2) in Capan-1 and HPAF-II, through lentiviral transduction. Cells transduced with shGFP were used as the control. HNF4A mRNA levels were assessed through RT-qPCR, normalized to GAPDH and β-actin levels and results were expressed as mean ± SEM compared to Capan-1 cells transduced with shGFP (set as 1).

| 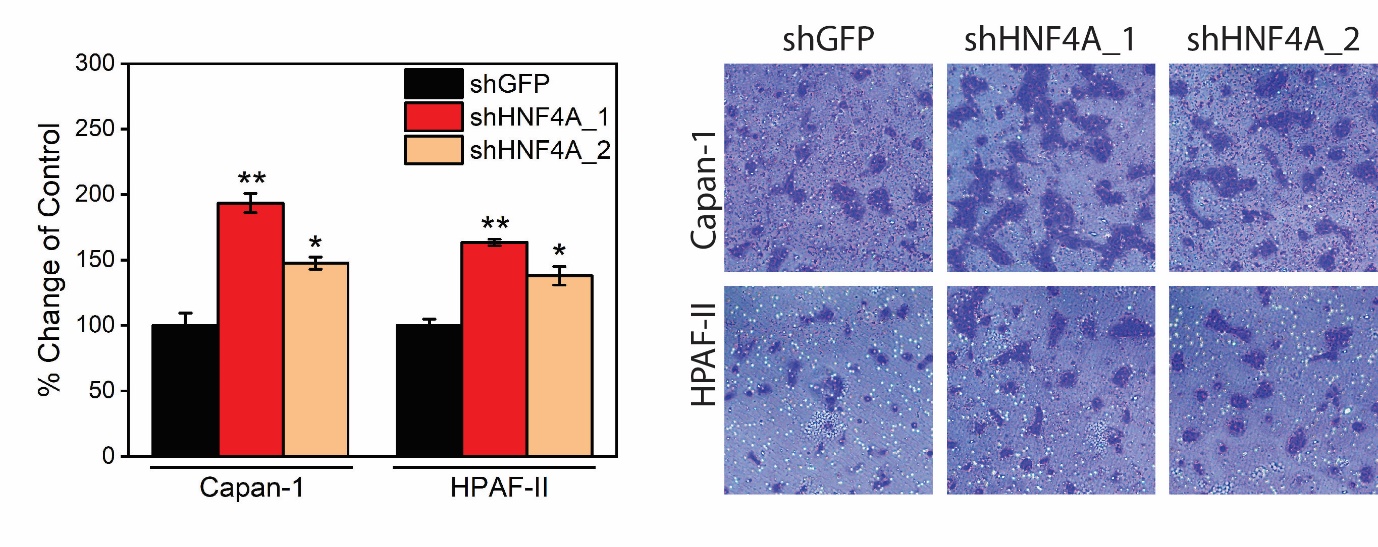 |
| --- |

**Figure A20:** HNF4A was stably suppressed, using two different shRNAs (shHNF4A_1 and _2) in Capan-1 and HPAF-II cells. Cells transduced with shGFP were used as the control. Invasion through matrigel was performed using transwell filter chambers and serum as the chemoattractant. 24h later invading cells were quantified, and results were expressed as the mean percentage change ± SEM of control invading cells (set as 100). Representative images were acquired at a 20X magnification, using an Evos microscope. Asterisks denote statistically significant differences, * *P* < .05, ** *P* <.01, Student’s t test.

| **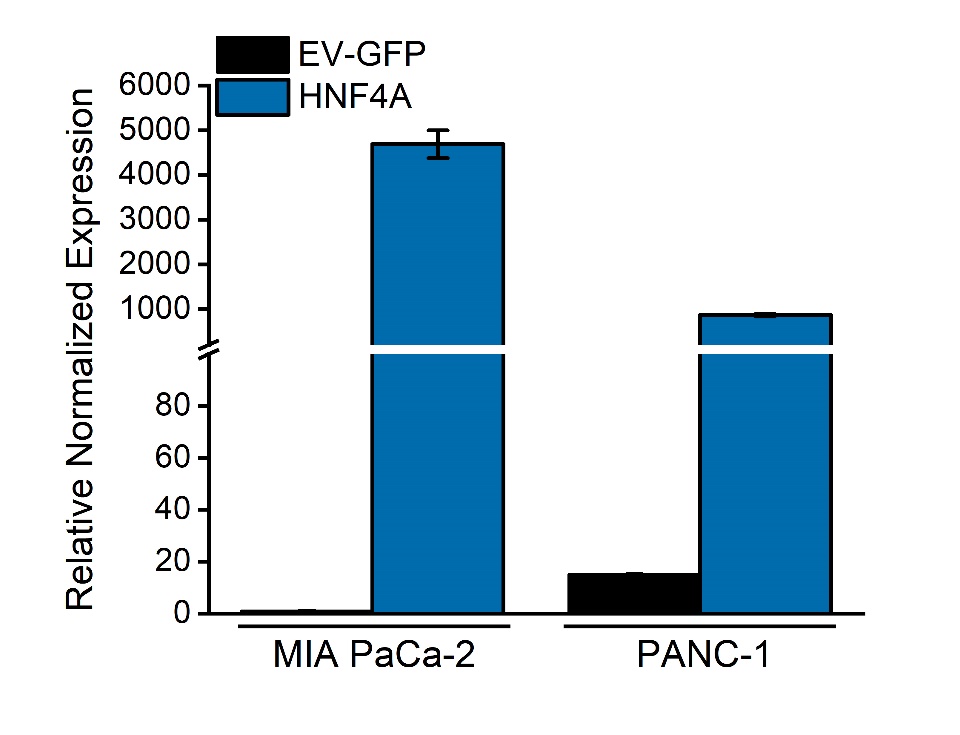** |
| --- |

**Figure A21:** Stable HNF4A overexpression was established through lentiviral transduction, in MIA PaCa-2 and PANC-1 cells. Cells transduced with the empty lentiviral vector, tagged with GFP (EV-GFP), were used as the control. HNF4A mRNA levels were assessed through RT-qPCR, normalized to GAPDH and β-actin levels and results were expressed as mean ± SEM compared to MIA PaCa-2 cells transduced with EV-GFP (set as 1).

| **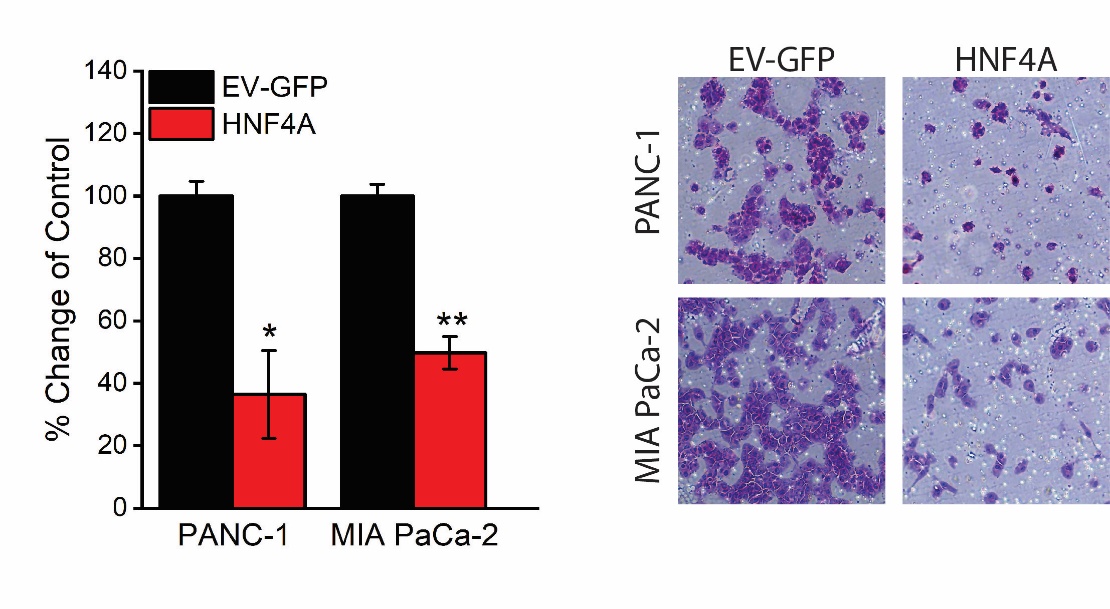** |
| --- |

**Figure A22:** Stable HNF4A overexpression was established through lentiviral transduction, in MIA PaCa-2 and PANC-1 cells. Cells transduced with the empty lentiviral vector, tagged with GFP (EV-GFP), were used as the control. Invasion through matrigel was performed using transwell filter chambers and serum as the chemoattractant. 24h later invading cells were quantified, and results were expressed as the mean percentage change ± SEM of control invading cells (set as 100). Representative images were acquired at a 20X magnification, using an Evos microscope. Asterisks denote statistically significant differences, * *P* < .05, ** *P* < .01, Student’s t test.

| **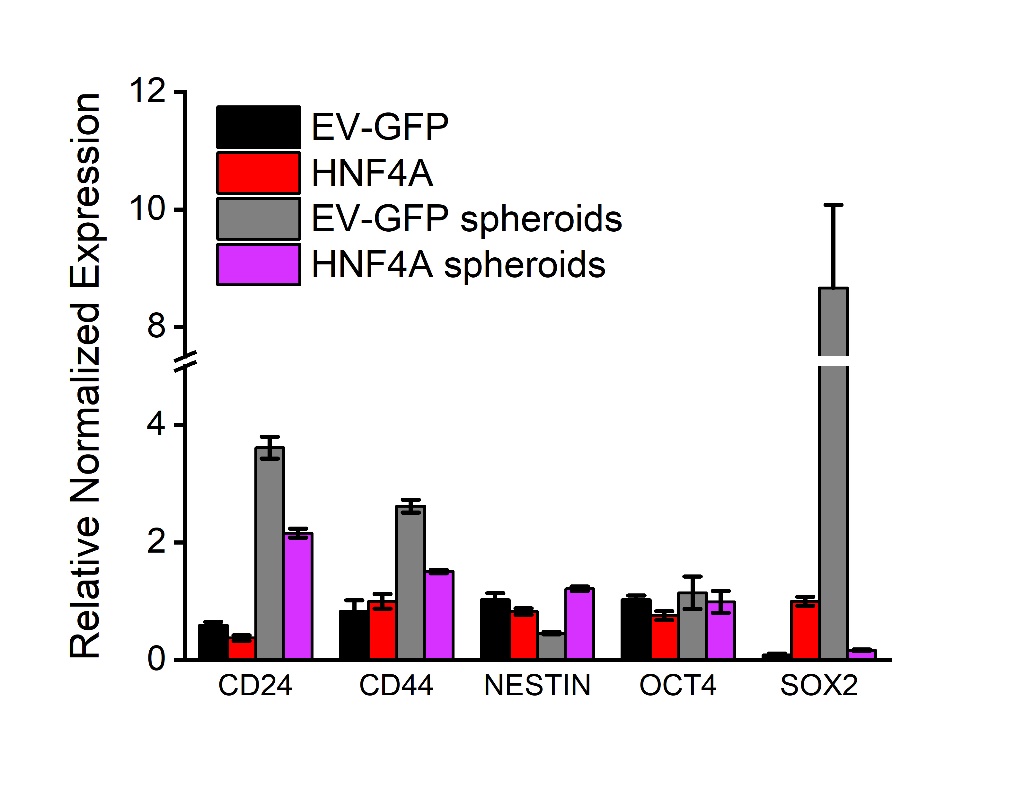** |
| --- |

**Figure A23:** Expression of cancer stem cell markers in adherent PANC‑1 cells and spheroids. Stable HNF4A overexpression was established through lentiviral transduction, in PANC-1 cells. Cells transduced with the empty lentiviral vector, tagged with GFP (EV-GFP), were used as the control. For the generation of spheroids, 1,000 cells were seeded in ultra-low attachment plates for 7 days. RNA was isolated from adherent cells (EV-GFP and HNF4A) and the respective spheroids and RT-qPCR was performed to determine the expression of CD24, CD44, NESTIN, OCT4 and SOX2.

**SUPPLEMENTARY TABLES**

**Table A1.** Molecular tools used for HNF4A expression manipulation and assessment.

| **Primers** | | | | **Sequence (5’-3’)** | | **Tm (^o^C)** | | **Product Size** | **Detected Isoforms** | | |
| --- | --- | --- | --- | --- | --- | --- | --- | --- | --- | --- | --- |
| HNF4A | Forward | | | TGTCCCGACAGATCACCTC | | 59.60 | | 121bp | HNF4alpha1-12 | | |
|  | Reverse | | | CACTCAACGAGAACCAGCAG | | 59.62 | |  |  |  |  |
| P1aF | Forward | | | ACATGGCCGACTACAGTGCT | | 60.74 | | 114bp | HNF4alpha1, 2 and 3 | | |
| P1R | Reverse | | | TGAGGTTGGTGCCTTCTGAT | | 60.66 | |  |  |  |  |
| P1bF | Forward | | | TCCGACATCACTGGAGCATA | | 60.22 | | 81bp | HNF4alpha4, 5 and 6 | | |
| P1R | Reverse | | | TGAGGTTGGTGCCTTCTGAT | | 60.66 | |  |  |  |  |
| P2aF | Forward | | | CCAGTGGAGAGTTCTTACGACA | | 59.40 | | 85bp | HNF4alpha7, 8 and 9 | | |
| P2R | Reverse | | | CACACAGGGCGCTGACAC | | 62.67 | |  |  |  |  |
| P2bF | Forward | | | AGGAGGAGGATGTCGGACTG | | 61.61 | | 115bp | HNF4alpha10, 11 and 12 | | |
| P2R | Reverse | | | CACACAGGGCGCTGACAC | | 62.67 | |  |  |  |  |
| **siRNAs** | | | | | **Targeted HNF4A sequence** | | **Targeted Isoforms** | | | **Cat No** | |
| siHNF4A_1 | | siRNA for HNF4A, #1 | | | Exon 7 in HNF4alpha2, 977nt | | HNF4alpha1-12 | | | s6696 Ambion | |
| siHNF4A_2 | | siRNA for HNF4A, #2 | | | Exon 4 in HNF4alpha2, 533nt | | HNF4alpha1-12 | | | s6698, Ambion | |
| siControl | | Non-targeting siRNA | | | N/A | |  | | | 4390846, Ambion | |
| **shRNAs** | | | | | **Targeted sequence** | | **Location** | | | **Targeted Isoforms** | |
| shHNF4A_1 | | | shRNA for HNF4A, #1 in pLKO.1 puro | | TGAGTATGCCTACCTCAAA | | Exon 6 in HNF4alpha2, 970-989nt | | | HNF4alpha1-12 | |
| siHNF4A_2 | | | shRNA for HNF4A, #2 in pLKO.1 puro | | TCACCTGATGCAGGAACATAT | | Exon 9 in HNF4alpha2, 1289-1310nt | | | HNF4alpha1, 2, 4, 5, 7, 8, 10, 11 | |
| shGFP | | | shRNA for GFP pLKO.1 puro | | AAGCTGACCCTGAAGTTCAT | | N/A | | | N/A | |
| **Antibody** | | | | | **Targeted HNF4A motif** | | **Recognised Isoforms** | | | | **Cat No** |
| HNF4-alpha (C11F12) Rabbit mAb | | | | | Proprietary, C-terminal region | | HNF4alpha1, 2, 4, 5, 7, 8, 10, 11 | | | | 3113S, Cell Signaling Technology |

**Table A2.** Genomic *loci* and genomic sequences analysed by bisulfite sequencing.

| **Regions** | **Locus (hg19)** | **Sequence** |
| --- | --- | --- |
| **Region 1** | chr20: 42984210-42984510 | CGCGGGTTCCCTAAGTGACTGGTTACTCTTTAACGTATCCACCCACCTTGGGTGATTAGAAGAATCAATAAGATAACCGGGCGGTGGCAGCTGGCCGCACTCACCGCCTTCCTGGTGGACGGGCTCCTGGTGGCTGTGCTGCTGCTGTGAGCGGGCCCCTGCTCCTCCATGCCCCCAGCTCTCCGG |
| **Region 2** | chr20: 43035886-43036186 | GAGGAGGAAGTTGTGTCTTCTCCATCCAACCATCCAAAGCCCTCCCCAGATTTAGCCGGCAGTGCGTGGTGGACAAAGACAAGAGGAACCAGTGCCGCTACTGCAGGCTCAAGAAATGCTTCCGGGCTGGCATGAAGAAGGAAGGTGAGCCTCGGCCCTCCCCGCCCCACCACCA |
| **Region 3** | chr20: 42984019-42984349 | CTCTCACCTCTCCAGCCCCTTCTGCTCCGGCCCTGTCCTCAAATTGGGGGGCTGATGTCCCCATACACCTGGCTCTGGGTTCCCCTAACCCCAGAGTGCAGGACTAGGACCCGAGTGGACCTCAGGTCTGGCCAGGTCGCCATTGCCATGGAGACAGCAACAGTCCCCAGCCGCGGGTTCCCTAAGTGACTGGTTACTCTTTAACG |
| **Region 4** | chr20:  42984366-42984666 | TGAGCGGGCCCCTGCTCCTCCATGCCCCCAGCTCTCCGGCTGGGTGGGCTTGGCCATGGTCAGCGTGAACGCGCCCCTCGGGGCTCCAGTGGAGAGTTCTTACGGTAAGTGGGGCTGGGGGAAGACTGGACAGGGCGGGACTGCGGTCAGCTTTGGGAGGCCATGGGACACCTCCCCGTGTGTTTCTTACGGGCCCAAAGCTCCTCCTGGAGCT |
| **Region 5** | chr20: 42984728-42985028 | GAAGCACCGTCCTGTTTCGATGCGGGGCAAATTGAGGTCCACCAGGAGAGGCTTGCTGGGCCTAGGTCACGTTGCTGGTGCATTATCAAGCTGGGCGTGGGACCGGGCAGCTCGGTCGCTCCGCACCTCCGTTGGCTCTGGATAATGGGGAGGAGGTAGAAAGCGCCGCGCAGGGTGGAGCGTTGGAAAATGAGAATATCTTGTGCGGACG |
| **Region 6** | chr20: 42983770-42984070 | ACTAGCAGTGAGACTCCAGGCATGCAATTTCTCTCTGTCCTTCAGTCCCTTCATCTCAAGGTTTAATTTAAATATGGTAACGCCTGTATGCAACTCCCAGCATCCAGTAGGCACTCACTAAACACAGTTCTCCACCCTCCTTTTTTCCTCTGCCCCTCCCTCGGTTTTCCCACTACT |
| **Region 7** | chr20: 43029606-43030006 | AACATCGGTGAGTTAGGGCCCCAGCAGTTGTAATTAGCACCCCGGGTGTCAGCCAGAAACCAACAAACAGCCAAATCCCTGCAGCCCCGCCCAGCCTATCCACCGGCGGGGGACCGATTAACCATTAACCCCCACCCCTCCCCGGCAGAGCCTCCACCCCTTCACAGAGGCTAGGCCAAGACTCCCAGCAGATCTTCCCAGAGGACGGTTTGAAAGGAAGG |

**Table A3.** Primers used for RT-qPCR.

| **Primers** | | **Sequence (5’-3’)** | **Tm (^o^C)** | **Product Size** |
| --- | --- | --- | --- | --- |
| GAPDH | Forward | ATGTTCGTCATGGGTGTGAA | 59.81 | 89bp |
|  | Reverse | GGTGCTAAGCAGTTGGTGGT | 60.18 |  |
| Beta-Actin | Forward | CCCAGCACAATGAAGATCAA | 59.65 | 103bp |
|  | Reverse | ACATCTGCTGGAAGGTGGAC | 60.12 |  |
| NESTIN | Forward | CTTCCCTCAGCTTTCAGGAC | 59.01 | 83bp |
|  | Reverse | TGGGAGCAAAGATCCAAGAC | 60.20 |  |
| OCT4 | Forward | AGTGAGAGGCAACCTGGAGA | 59.99 | 125bp |
|  | Reverse | GCCGGTTACAGAACCACACT | 60.04 |  |
| SOX2 | Forward | CCTCTTCCTCCCACTCCAG | 59.77 | 120bp |
|  | Reverse | GGGACATGTGAAGTCTGCTG | 59.26 |  |
| CD44 | Forward | AAGGTGGAGCAAACACAACC | 60.01 | 115bp |
|  | Reverse | ACTGCAATGCAAACTGCAAG | 60.06 |  |
| CD24 | Forward | ACCCACGCAGATTTATTCCA | 60.33 | 110bp |
|  | Reverse | CCTTGGTGGTGGCATTAGTT | 59.85 |  |
